# Conventional wisdom on roosting behavior of Australian flying‐foxes—A critical review, and evaluation using new data

**DOI:** 10.1002/ece3.8079

**Published:** 2021-09-09

**Authors:** Tamika J. Lunn, Peggy Eby, Remy Brooks, Hamish McCallum, Raina K. Plowright, Maureen K. Kessler, Alison J. Peel

**Affiliations:** ^1^ Centre for Planetary Health and Food Security Griffith University Nathan QLD Australia; ^2^ School of Biological Earth and Environmental Sciences University of New South Wales Sydney NSW Australia; ^3^ Department of Microbiology and Immunology Montana State University Bozeman MT USA; ^4^ Department of Ecology Montana State University Bozeman MT USA

**Keywords:** camp, conservation, fruit bat, habitat, management, Pteropodidae

## Abstract

1. Fruit bats (Family: Pteropodidae) are animals of great ecological and economic importance, yet their populations are threatened by ongoing habitat loss and human persecution. A lack of ecological knowledge for the vast majority of Pteropodid species presents additional challenges for their conservation and management.
2. In Australia, populations of flying‐fox species (Genus: *Pteropus*) are declining and management approaches are highly contentious. Australian flying‐fox roosts are exposed to management regimes involving habitat modification, through human–wildlife conflict management policies, or vegetation restoration programs. Details on the fine‐scale roosting ecology of flying‐foxes are not sufficiently known to provide evidence‐based guidance for these regimes, and the impact on flying‐foxes of these habitat modifications is poorly understood.
3. We seek to identify and test commonly held understandings about the roosting ecology of Australian flying‐foxes to inform practical recommendations and guide and refine management practices at flying‐fox roosts.
4. We identify 31 statements relevant to understanding of flying‐fox roosting structure and synthesize these in the context of existing literature. We then contribute a contemporary, fine‐scale dataset on within‐roost structure to further evaluate 11 of these statements. The new dataset encompasses 13‐monthly repeat measures from 2,522 spatially referenced roost trees across eight sites in southeastern Queensland and northeastern New South Wales.
5. We show evidence of sympatry and indirect competition between species, including spatial segregation of black and grey‐headed flying‐foxes within roosts and seasonal displacement of both species by little red flying‐foxes. We demonstrate roost‐specific annual trends in occupancy and abundance and provide updated demographic information including the spatial and temporal distributions of males and females within roosts.
6. Insights from our systematic and quantitative study will be important to guide evidence‐based recommendations on restoration and management and will be crucial for the implementation of priority recovery actions for the preservation of these species in the future.

## Graphical abstract

Pteropodid bats are in decline globally. A major challenge for these species is that policies for conservation (roost restoration) and conflict management (roost modification or destruction) are often in direct contrast. Additionally, systematically informed, baseline ecological knowledge is limited for the vast majority of these species, so the impact and effectiveness of efforts to contribute to either conservation or conflict goals are unknown. This study is a systematic and multifaceted investigation into the roosting ecology of Australian flying‐foxes that aims to identify and empirically evaluate commonly held but anecdotal understandings about the ecology of these species, to contextualize management prescriptions in the context of species ecology.

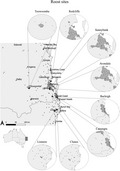

## 1 INTRODUCTION

Fruit bats (Family: Pteropodidae) are animals of extraordinary ecological and economic importance (Fujita & Tuttle, 1991). As long‐distance seed dispersers and pollinators, fruit bats play a crucial role in the maintenance and regeneration of forest ecosystems (Hodgkison et al., 2003; Oleksy et al., 2015; Shilton et al., 1999). Moreover, fruit bats are responsible for the propagation of at least 289 plant species across their distribution, 186 of which have economic value, making fruit bats important contributors to the sustainability of human livelihoods (Fujita & Tuttle, 1991). Despite their importance, many fruit bat species are in severe decline. Half are listed as near threatened to extinct according to the IUCN (88 of the 177 species with sufficient data) (IUCN, 2020), with human persecution and habitat loss identified as two of the largest threats imposed on these species (Acharya et al., 2011; Andrianaivoarivelo et al., 2011; IUCN, 2020; Jenkins et al., 2007). While measures have been taken in some countries to reverse this trend—including increased legislative protection (Aziz et al., 2016; Eby & Lunney, 2002a; Thiriet, 2010) and community awareness campaigns (Anthony et al., 2018; Carroll & Feistner, 1996; Trewhella et al., 2005)—conservation and management efforts for the majority of these species remain hindered by an enduring absence of ecological knowledge (Fujita & Tuttle, 1991; Mickleburgh et al., 2002) and ongoing conflict with humans (Aziz et al., 2016; Currey et al., 2018).

These same conservation challenges persist for Australian flying‐foxes (Genus: *Pteropus*) despite improved levels of protection. Indiscriminate and widespread persecution and killing of flying‐foxes were persistent until the ~1990s (Fujita & Tuttle, 1991; Hall, 2002; Ratcliffe, 1931). Species listed as threatened are now afforded national protection under the Environment Protection and Biodiversity Conservation Act 1999 (EPBC Act) (Department of Agriculture Water & the Environment, 1999), and other species are protected from harm under state‐level native species legislations (Department of Environment & Primary Industries State Government of Victoria, 1988; New South Wales Government, 2016; Queensland Government, 1992). However, loss and degradation of roosting habitat continues to pose a substantial threat, and management of these species must additionally balance conservation outcomes with negative public perception and human–wildlife conflict (e.g., BBC News Australia, 2017; Kohut, 2017; Welle, 2021).

A major challenge for these species is that policies for conservation and conflict management are often in direct contrast. The identification, management, and protection of roosting habitat are listed as priority recovery actions for the vulnerable grey‐headed flying‐fox (*Pteropus poliocephalus*) and endangered spectacled flying‐fox (*Pteropus conspicillatus*) (Commonwealth of Australia, 2017a). Yet in direct contrast, roost management policies and guidelines that aim to reduce human–wildlife conflict often promote removal of roost trees to create perimeter buffers between the roost and private properties, which can exceed 50 meters in some cases (State of NSW & Office of Environment & Heritage, 2018). In more extreme cases, flying‐fox roost management permits can be granted to disturb, drive away, or destroy flying‐fox roosts entirely (Lenson, Mo, Roache, et al., 2020; Mo et al., 2020).

Management challenges in Australia are being further compounded by an emerging and accelerating trend of urbanization of flying‐fox roost sites, and fragmentation of roost populations (Meade et al., 2021; Tait et al., 2014; Williams et al., 2006). Roost structures are transitioning from large roosts that are seasonally occupied by nomadic individuals into smaller, continuously occupied roosts in urban areas (Van der Ree et al., 2006; Eby et al. in review). This fragmentation, or fissioning, of roost populations has been attributed to environmental change ‐ both land clearing of winter flowering native species in southeastern Australia (Eby et al., 1999) and the concurrent increase in availability of exotic winter food resources in urban areas (Parry‐Jones & Augee, 2001; Williams et al., 2006). As a consequence, increasing numbers of roosts have formed near residential housing, particularly in metropolitan areas such as Sydney, the Gold Coast, and Brisbane, despite overall population declines (Tait et al., 2014). These urban roosts often develop into sites of ongoing conflict with neighbors (Commonwealth of Australia, 2017b), and there has been growing demand to reduce the impact of roosts on local communities through active management of flying‐fox camps (Currey et al., 2018). Similar changes with fragmentation and urbanization have been observed elsewhere (Hahn, Epstein, et al., 2014; Hahn, Gurley, et al., 2014; Peel et al., 2017), suggesting that this occurrence is likely representative of other systems across the range of Pteropodids.

A second major challenge for management of these species is that systematically informed, baseline ecological knowledge is limited, so the impact and effectiveness of efforts to contribute to either conservation (roost restoration) or conflict (roost modification) goals are unknown. Roosting requirements of these species are not well understood (Commonwealth of Australia, 2017a) beyond broadscale trends in roosting patterns (e.g., Tidemann et al., 1999; Vardon & Tidemann, 1999), migration (Eby, 1991; Eby et al., 1999; Meade et al., 2021), and studies on sociality and behavior (Klose et al., 2009; Nelson, 2020, 1965b; Welbergen, 2005). Detailed (fine‐scale) spatiotemporal patterns in animal density and tree use remain unquantified (Commonwealth of Australia, 2017a), and knowledge on historical usage patterns (e.g., Nelson, 2020, 1965b; Ratcliffe, 1931; Tidemann et al., 1999; Vardon & Tidemann, 1999) may be inconsistent with current usage patterns. This lack of detailed information is of particular concern, as current conservation strategies that aim to identify, protect, and restore important roosting habitat, and practices for managing conflict, are necessarily founded on observations that may not fully reflect the habitat requirements of the animals. In this context, the number of flying‐fox roosts exposed to programs of vegetation modification is increasing rapidly in Australia, yet the potential impact of modifications to roosting habitat on flying‐foxes is largely unknown. More information is needed to provide baseline ecological data in this time of rapid ecological change, and to guide and support vegetation management practices and decision‐making criteria to provide a realistic representation of the roosting habitat needs and preferences of flying‐foxes. Systematic and comprehensive examination of multiple species in Australia may also help identify whether generalities exist among Pteropodids, and guide understanding in systems where more limited data and resources are available.

In this paper, we seek to identify and evaluate commonly held understandings about the roosting ecology of Australian flying‐foxes, focusing on species on the Australian mainland. We first review “gray literature” (management, recovery, and restoration plans or reports published by state government and local groups) to identify commonly held understandings concerning flying‐fox roosting structure. We then review the existing empirical literature, to critically evaluate the extent of empirical support for these statements and highlight gaps in empirical evidence. Lastly, we utilize high‐resolution spatial mapping techniques and monthly field observations to systematically and quantitatively document spatial and intra‐annual temporal patterns in flying‐fox roost and tree use in southeast Queensland and northeast New South Wales. This approach allows us to highlight where quantitative information on flying‐fox roosting has been missing, and where updated information may be required. Our new dataset is the first to capture fine‐scale spatial and temporal dynamics of flying‐fox roost use in a structured, repeatable design, and provides baseline information in a time of rapid ecological change. Such a systematic and quantitative study will be important for informing evidence‐based recommendations to guide vegetation modification practices and improve roost management strategies for flying‐fox conservation. This will be crucial for implementation of effective habitat restoration projects, to successfully balance the management of these threatened, contentious, and urbanizing wildlife, and to guide comparable approaches in other Pteropodid species across their range.

## 2 METHODS

Four species of flying‐fox occur in Australia. These include the grey‐headed flying‐fox (*P*. *poliocephalus*), black flying‐fox (*Pteropus alecto*), spectacled flying‐fox (*P. conspicillatus*), and little red flying‐fox *(Pteropus scapulatus*). Little red flying‐foxes are the smallest of the species, ranging in adult weight from approximately 330–550g and with a wingspan of around 0.9 m (Bartholomew et al., 1964). Adult weight of the other species each range between 650 and 1,000 g, and wingspan is around 1.2 m (Eby & Lunney, 2002b). Reproduction is seasonal and synchronous, with each species showing a single birth pulse per year—typically October–November for grey‐headed and black flying‐foxes, and April–May for little red flying‐foxes (Eby & Lunney, 2002b; McIlwee & Martin, 2002).

Flying‐foxes are highly gregarious and occur in large communal aggregations known as “roosts” or “camps” (Ratcliffe, 1931). Flying‐foxes roost in the exposed branches of trees, and a single roost community can collectively number hundreds to hundreds of thousands of individuals (National Flying‐Fox Monitoring Program, 2017, 1965a). Roosts are used as daytime rest stops by animals that forage in surrounding areas or as short‐term stopover sites by migrating animals and function as maternity colonies in the breeding season (Eby & Palmer, 1991; Tidemann & Nelson, 2004). The locations of roosts are generally stable through time (e.g., some roosts have documented histories that exceed 100 years) (Lunney & Moon, 1997), though patterns of camp occupation can vary and include roosts that are inhabited continuously, seasonally, or irregularly (Parry‐Jones & Augee, 2001).

### 2.1 Review of gray literature

Flying‐fox management is generally undertaken in line with site‐specific roost management plans (e.g., Council of Ipswich, 2016; EcoLogical, 2014; Scenic Rim Regional Council, 2015), which are adopted by local government councils based on their state's flying‐fox camp management policy (e.g., Queensland: SEQ Catchments (2012), State of Queensland Department of Environment and Science (2020); and New South Wales: State of NSW and Office of Environment and Heritage (2018)). We focused on statements made in state‐level documents, as these are the primary resource for individual roost plans. We identified common statements/understandings across these documents, with particular emphasis on those that pertain to (1) routine vegetation management activities (weed removal and trimming understory vegetation); (2) creation of buffers (either by clearing/trimming canopy trees, or disturbing animals at the roost boundary); and (3) restoration interventions.

### 2.2 Review of existing empirical support

We conducted a systematic literature search of peer‐reviewed published literature using ISI’s Web of Knowledge (27 July 2020). Keywords were chosen to target studies evaluating the within‐ and between‐roost structure of Australian flying‐foxes (Table S1). This included any studies relevant to (1) the physical structure of roosts (e.g., area, tree structure, tree/roost selection), (2) the social structure of roosts (e.g., demographic and species structuring), (3) roosting behavior (e.g., territoriality and fidelity of individuals), (4) movement and migration relating to occupancy and abundance of roosts, and (5) roost microclimate. In addition to the literature search, reference lists and relevant studies already known to the authors were also screened to identify potentially relevant studies not captured by our initial search. We also included empirical support from key unpublished sources (e.g., dissertations).

### 2.3 Empirical data collection

**FIGURE 1:**
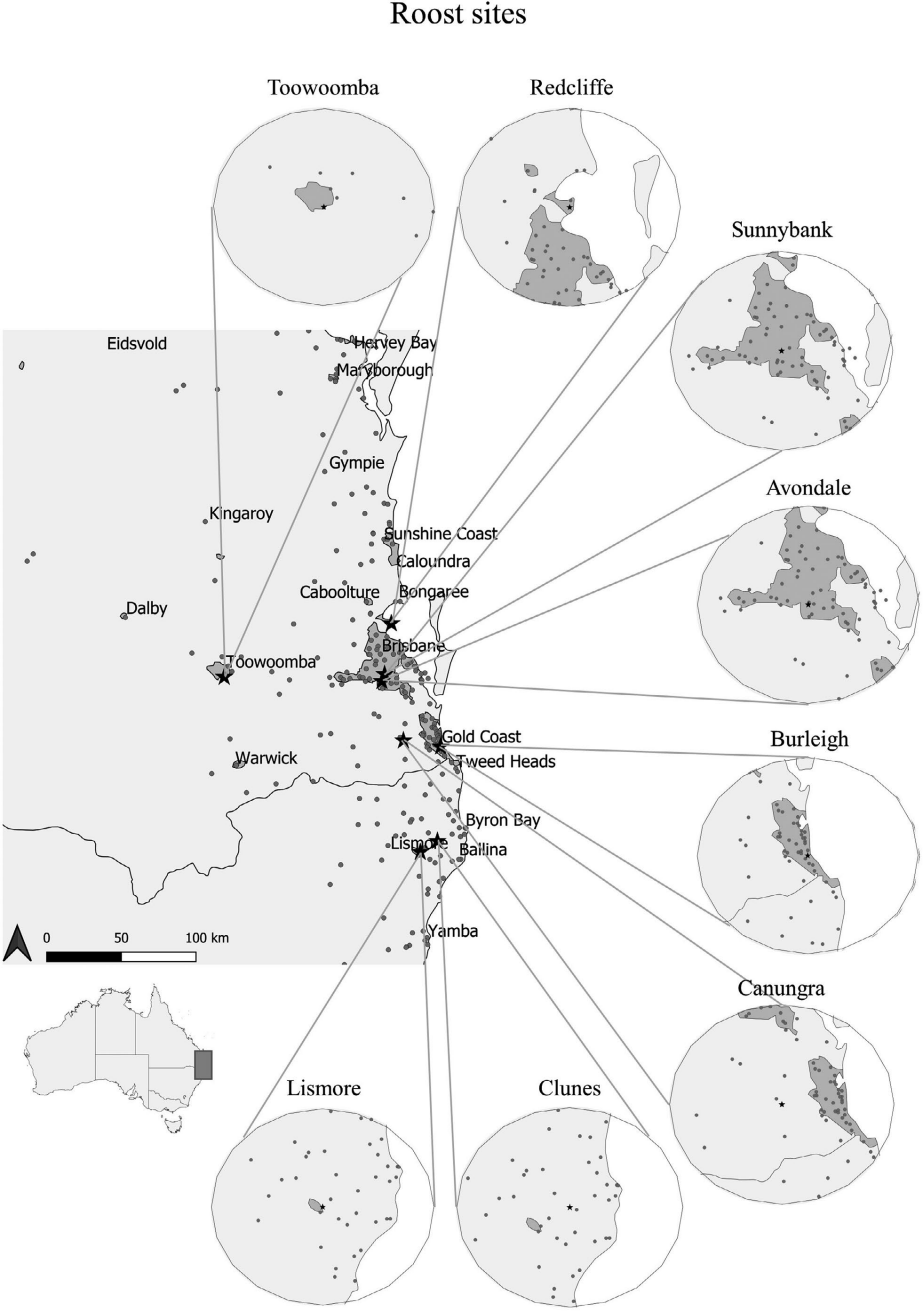
Map of roost sites included in the study. Gray shading indicates urban land cover of dense human habitation (as per Schneider et al., 2009), and gray circles are locations of flying‐fox roosts. Circles show 45‐km foraging radii from roost study sites (as per Giles et al., 2018). GIS land‐cover data were downloaded from Natural Earth (2020, 1965b) and flying‐fox roost locations obtained from the National Flying‐Fox Monitoring Program (2017, 1965a)

We collected data on roosting structure at eight sites in southeast Queensland and northeast New South Wales (Figure 1). These sites were chosen to represent a gradient of habitats utilized by flying‐foxes, ranging from metropolitan areas of Brisbane and the Gold Coast, to roosts in peri‐urban and rural areas (Figure 1, Table 1). All sites were previously documented as having a continuous population of grey‐headed or black flying‐foxes. Little red flying‐foxes visited some roost sites intermittently; however, no roost sites occurred within the distribution of spectacled flying‐foxes (National Flying‐Fox Monitoring Program, 2017, 1965a).

**TABLE 1:** Information on roost sites included in the study

Roost site	Type	Year of formation/new overwintering	Number of neighboring roosts within foraging radius	Area of urban land use within foraging radius (km^2^)	Distance to nearest urban edge (km)	Number of tagged trees inside subplots (number of trees per km^2^)
	*Within 45‐km foraging radius*					
Toowoomba		2009	9	135.2	0	118 (29.5)
Redcliffe		2003	41	1,447.3	0	286 (71.5)
Sunnybank	Contemporary	2009	67	1,650.0	0	226 (56.5)
Avondale	Contemporary	2008	68	1,650.0	0	268 (67)
Burleigh	Contemporary	2013	50	1601.6	0	327 (81.8)
Canungra		1996	59	1601.6	11.5	474 (118.5)
Clunes		2014	40	20.8	14.4	349 (87.3)
Lismore		2007	37	20.8	0.4	474 (118.5)

Note

Contemporary roost types (features grayed) are characterized by: being a new overwintering site (defined as having either formed since 2007 or changed to an overwintering site since 2007), having a high number of neighboring roosts within 45 km, having a high proportion of surrounding urban land cover, and by being in close proximity to urban land cover. Foraging radii enclose 45 km from roost study sites (as per Giles et al., 2018). Data are from the National Flying‐Fox Monitoring Program (2017, 1965a).

We mapped the spatial arrangement of all overstory, canopy, and midstory trees in a grid network of 10 stratified random subplots (20 × 20 meters each) per roost site. Subplots were stratified throughout perceived “core” (five subplots) and “peripheral” (five subplots) roosting areas, classed as areas observed to be frequently occupied (core) or infrequently (peripheral) by bats (Welbergen, 2005). Core and peripheral areas were evaluated from regular observations made prior to roost tree mapping, though note that these categories were revised subsequently with the quantitative data. Trees were mapped and tagged using tree survey methods described in the “Ausplots Forest Monitoring Network, Large Tree Survey Protocol” (Wood et al., 2015). To evaluate spatiotemporal patterns in roosting, we revisited all tagged trees and scored the extent of species occupancy using the following tree abundance index: 0 = zero bats; 1 = 1–5 bats; 2 = 6–10 bats; 3 = 11–20 bats; 4 = 21–50 bats; 5 = 51–100 bats, 6 = 101–200 bats, and 7 = >200 bats. For a subset of trees (*N* = 60 per site, consistent through time), absolute counts and minimum/maximum roosting heights of each species were taken. Overall roost perimeter (perimeter of area occupied) was mapped with GPS (accurate to 10 m) immediately after the tree survey to estimate perimeter length and roost area. Total abundance at each roost was also estimated with a census count of bats where feasible (i.e., where total abundance was predicted to be <5,000 individuals), or by counting bats as they emerged in the evening from their roosts (“fly‐out”), as per recommendations in Westcott et al. (2011). If these counts could not be conducted, population counts from local councils (conducted within ~a week of the bat surveys) were used, as the total abundance of roosts is generally stable over short time frames (Nelson, 2020, 1965b). Because roost estimates become more unreliable with increasing total abundance, and because our estimation methods were intrinsically linked with total abundance, we converted the total estimated abundance into an index estimate (where bin ranges increase with total abundance) for use in analyses, as per values used by the National Flying‐Fox Monitoring Program (2017, 1965a). Index categories were as follows: 1 = 1–499 bats, 2 = 500–2,499 bats, 3 = 2,500–4,999 bats, 4 = 5,000–9,999 bats, 5 = 10,000–15,999 bats, 6 = 16,000–49,999 bats and 7 = >50,000 bats. Roosting surveys were repeated once a month for 13 months (August 2018–August 2019). More detailed methods of empirical data collection can be found in Appendix S1.

### 2.4 Statistical analyses

The main statistical comparisons tested with our empirical data were as follows: (1) whether density of occupation is greater for subplots in “core” areas of the roost compared with subplots in irregularly occupied “peripheral” areas (defined by occupation greater than or less than 80% of surveys respectively (Appendix S1); (2) whether bat occupation decreases with distance from the roost center (per species); (3) whether bat species segregate in vertical space; and (4) whether dominant individuals occupy the center of roosts, and subdominant individuals the outer area (per species). We also provide qualitative comparisons of (5) seasonal patterns of abundance and occupancy per species; and (6) whether bat species segregate in horizontal space. Because we do not observe dominance directly, we assume that only dominant reproducing males share their territory with females and their young and use the proportion of males per tree as a proxy for dominance structure in the roost. This is consistent with behavioral studies of dominance and observations of “bachelor male trees” containing entirely nonreproducing males (e.g., Markus, 2002 and Welbergen, 2005).

We utilized generalized additive models for all statistical comparisons to allow for nonlinearity, with random effects modeled with smooth functions. Roost site and subplot were modeled using a standard random‐effects smoothing function. Session was modeled using a cyclic cubic regression spline in cases where seasonality in the time series was evident (all comparisons except those involving the proportion of male black, male grey‐headed, and combined male bats per tree); otherwise, session was modeled with a standard random‐effects smoothing function. We accounted for nonindependence (nesting) of random effects by including an autoregressive model for errors in the model (Laurinec, 2017; Yang et al., 2012). For the comparisons involving evaluation of species, models were run separately for each species owing to differences in seasonality of occupation (and so differences in the fit of cyclic cubic regression splines). Error distribution for comparisons was specified according to data type and extent of zero‐inflation (as per Crawley, 2013). We fit the models and performed checks of standardized residuals in R (version 4.0.2), using the “mgcv” package (functions “gamm” and “gam.check”) (as per Wood, 2017). See Appendix S1 for more detailed information on modeling decisions, a summary table of comparisons, and a breakdown of spatial and temporal replicates of measures. Summarized data are given in Appendix S2, and annotated R code is available on GitHub at: https://github.com/TamikaLunn/FF‐roost‐ecology.

## 3 RESULTS

From our review of management, recovery, and restoration documents published by state government, we highlighted 31 commonly held understandings relevant to flying‐fox roosting structure (Table 2). From our systematic search for empirical literature, we generated a total of 79 search results. Of these, 52 were removed through screening (10 from outside the Australian mainland, 4 on non‐Pteropus species, and 38 focused on topics other than roost structure). An additional 18 published studies and 4 honors/PhD theses were included from citations and the author's reference collections, giving 49 included studies in total (Appendix S1). Lastly, we generated an empirical dataset consisting of 13‐monthly repeat measures from 2,522 trees across eight roost sites. Roost sites contained 118–474 measured and tagged trees each, with an average of 2 (sparsely structured) to 75 (densely structured) trees per 20x20 meter subplot. Tree roosting height and count were recorded for 9,056 trees out of 32,206 repeat measures. (Note that our total repeat measures were less than 32,786 owing to cases of tree removal through the duration of the survey.) We report model outputs of main interest in the main text, but see Appendix S3 for full model output.

**TABLE 2:** Common understandings in state‐level management documents

Understandings	Referenced by	Empirical evidence		Additional evidence (this study)	
		Support	Contradict	Support	Contradict
**Use of area**					
Some areas of permanent camps are more consistently occupied (“core areas”) than others	SEQ Catchments (2012); EcoLogical (2014)	Welbergen (2005); Richards (2002); Nelson (2020, 1965b)		Figure 2	
“Core areas” are more densely occupied than “peripheral areas”	SEQ Catchments (2012)	Nelson (2020, 1965b); Welbergen (2005)		Figure 3; Figure 4; Appendix S3	
Roost area fluctuates with total abundance	SEQ Catchments (2012); EcoLogical (2014)	Welbergen (2005); Pallin (2000); Larsen et al. (2002)		Figure 5	
Flying‐foxes adjust the location of “core areas” through time	SEQ Catchments (2012)	Hall (2002), Pallin (2000)	Welbergen (2005)		
Areas outside of the “core area” are used by more transient animals	SEQ Catchments (2012)	Welbergen (2005)			
**Spatial segregation of species**					
Species share roosts sites, but segregate spatially within	Commonwealth of Australia (2017a)	Welbergen (2005); Ratcliffe (1932); Parsons et al. (2010); Nelson (2020, 1965b); Klose et al. (2009)	Parsons et al. (2010); Markus (2002)	Figure 6; Appendix S4	
Large influxes of species into roosts (especially little red flying‐foxes) can displace other species		Birt and Markus (1999)		Appendix S4	
Species roost at different heights	Geolink (2010)	Welbergen (2005); Roberts (2005)		Figure 7	
Indirect competition favors black flying‐foxes over grey‐headed flying‐foxes	Commonwealth of Australia (2017a); EcoLogical (2014)	Ratcliffe (1931)	Markus (2002); Roberts (2005)		
**Demographic/social structure**					
The majority of roost trees are occupied by mixed groups of adults, with territories comprised of a single male and one or more females and their dependent young	SEQ Catchments (2012); State of Queensland Department of Environment and Science (2020)	Welbergen (2005); Puddicombe (1981); Nelson (2020, 1965b); Nelson (2017, 1965a); Markus and Blackshaw (2002); Markus (2002); Eby et al. (1999); McWilliam (1984); Connell (2003)	Welbergen (2005); Nelson (2020, 1965b); Nelson (2017, 1965a)	Figure 8	
Dominant individuals (defined as reproducing males and females) occupy the center of roosts and subdominant individuals (defined as nonreproducing males and females) the outer area	State of Queensland Department of Environment and Science (2020)	Nelson (2020, 1965b); Welbergen (2005)	Puddicombe (1981); Markus and Blackshaw (2002)		Figure 8; Appendix S5
Individuals at the periphery of groups act as “guards”	State of Queensland Department of Environment and Science (2020)	Nelson (2020, 1965b); Klose et al. (2009)			
Juveniles wean and leave their mothers from January and form groups on the edge of their existing roost or at another site	State of Queensland Department of Environment and Science (2020)		Welbergen (2005); Nelson (2020, 1965b); Nelson (2017, 1965a); Markus and Blackshaw (2002); Eby et al. (1999); Connell (2003)		
The roosting positions of individual males are highly consistent, and animals return to the same branch of a tree over many weeks or months	SEQ Catchments (2012)	Welbergen (2005); Markus and Blackshaw (2002); Markus (2002)	Tidemann and Nelson (2004); Roberts et al. (2012b); Parsons et al. (2011)		
**Roost abundance/occupancy**					
Individual roosts have distinguishable seasonal patterns of abundance and occupation.	*Abundance:* Commonwealth of Australia (2017a); *Occupation:* State of Queensland Department of Environment and Science (2020)	*Abundance* Westcott et al. (2018); Welbergen (2005); Tait et al. (2014); Parry‐Jones and Augee (2001); Parry‐Jones and Augee (1992); Nelson (2020, 1965b); Nelson (2017, 1965a); Meade et al. (2019) *Occupation* Welbergen (2005); Vardon and Tidemann (1999); Parry‐Jones and Augee (1992); Parry‐Jones (1985); Nelson (2020, 1965b); Nelson (2017, 1965a); Nelson (2020, 1965b); Nelson (2017, 1965a); Klose et al. (2009); Puddicombe (1981); Roberts (2005)	*Abundance* Shilton et al. (2008); Richards (2002); Roberts (2005) *Occupation* Van der Ree et al. (2006); Richards (2002); Puddicombe (1981); Shilton et al. (2008)	Figure 9	
Intra‐ and interannual variations in abundance can be extreme	Commonwealth of Australia (2017a)	Westcott and McKeown (2004); Tait et al. (2014); Welbergen (2008); Welbergen (2005); Vardon and Tidemann (1999); Ratcliffe (1931); Ratcliffe (1932); Eby (1991); Eby and Palmer (1991); Van der Ree et al. (2006); Eby and Lunney (2002a); Roberts et al. (2012a); Richards (2002); Parry‐Jones and Augee (2001); Parry‐Jones and Augee (1992); Pallin (2000); Meade et al. (2019); Loughland (1998); Giles et al. (2016); Forsyth et al. (2006); Eby et al. (1999); Lunney and Moon (1997)	Roberts (2005)	Figure 9	
Roost abundance peaks in March	State of Queensland Department of Environment and Science (2020)	Van der Ree et al. (2006); Tait et al. (2014); Meade et al. (2019); Eby (1991); Eby and Palmer (1991); Nelson (2017, 1965a)	Westcott et al. (2018); Welbergen (2005); Vardon and Tidemann (1999); Vardon et al. (2001); Roberts et al. (2012a); Richards (2002); Parry‐Jones and Augee (2001); Parry‐Jones and Augee (1992); Pallin (2000) (citing personal communication with M. Beck), Nelson (2020, 1965b), Nelson (2017, 1965a)		Figure 9; Appendix S3
Consistent (interannual) patterns in abundance and use are more commonly observed in roosts located in (1) extensive areas of rainforest and (2) urban areas	SEQ Catchments (2012), Commonwealth of Australia (2017a)	*Extensive rainforest* Parry‐Jones (1985) *Urban areas* Tait et al. (2014), Welbergen (2005), Van der Ree et al. (2006), Richards (2002), Williams et al. (2006), Parry‐Jones and Augee (2001), Parry‐Jones and Augee (1992)			
**Habitat preferences**			Stager and Hall (1983)		
The habitat patch must be at least 1ha in size but be large enough to accommodate and sustain large numbers of flying‐foxes. For a small roost (10,000 bats), the area needed is approximately 3ha, and for a large roost (50,000), the area needed is 10ha	SEQ Catchments (2012), State of NSW and Department of Planning Industry and Environment (2019) as per State of NSW and Office of Environment and Heritage (2018), EcoLogical (2014)	Pallin (2000), Roberts (2005)			
Flying‐foxes prefer complex vegetation structure (upper, mid‐, and understory layers)	SEQ Catchments (2012), State of NSW and Department of Planning Industry and Environment (2019) as per State of NSW and Office of Environment and Heritage (2018)	Pallin (2000) (citing report by Buchanan)			
Flying‐foxes prefer dense vegetation	SEQ Catchments (2012)	Roberts (2005)			
Flying‐foxes prefer a dense understory	SEQ Catchments (2012)	Roberts (2005)			
Flying‐foxes prefer a closed canopy at least 3−5 m high	SEQ Catchments (2012), State of NSW and Department of Planning Industry and Environment (2019) as per State of NSW and Office of Environment and Heritage (2018), EcoLogical (2014)	Tidemann et al. (1999), Tidemann (1999), Roberts (2005)	Welbergen (2005)		
The structure of roost‐wide vegetation is more important than the characteristics of individual roost trees (e.g., species, canopy cover)	SEQ Catchments (2012)	Palmer and Woinarski (1999), Pallin (2000), Vardon et al. (2001), Tidemann et al. (1999), Vardon and Tidemann (1999), Hall and Richards (2000), Roberts (2005)			
Flying‐foxes prefer level topography (<5° incline)	SEQ Catchments (2012), State of NSW and Department of Planning Industry and Environment (2019) as per State of NSW and Office of Environment and Heritage (2018)	Roberts (2005)			
Flying‐foxes prefer to roost within 50 km of the coastline or at an elevation <65 m above sea level	SEQ Catchments (2012), State of NSW and Department of Planning Industry and Environment (2019) as per State of NSW and Office of Environment and Heritage (2018)	Hall and Richards (2000), Roberts (2005)	Ratcliffe (1931), Ratcliffe (1932)		
**Roost macroclimate**					
The midstory vegetation within roosts is critical for maintaining a cool, humid, and sheltered environment that is stable against the outside environment	SEQ Catchments (2012), State of NSW and Department of Planning Industry and Environment (2019) as per State of NSW and Office of Environment and Heritage (2018)	Loughland (1998)	Snoyman and Brown (2010)		
**Negative impacts from flying‐foxes**					
Impacts sustained over several years of flying‐fox occupancy can lead to damage and/or death of individual roost trees	SEQ Catchments (2012), State of Queensland Department of Environment and Science (2020)	Welbergen (2005), Richards (2002), Pallin (2000), McWilliam (1984), Hall (2002)			
Some tree species are more resilient to damage by flying‐fox roosting than others	SEQ Catchments (2012)				
In small remnant patches, the process of opening the canopy (from tree damage by roosting) will increase the impact of invasive weeds	SEQ Catchments (2012), State of Queensland Department of Environment and Science (2020)	Pallin (2000), McWilliam (1984), Hall (2002)			
Where sufficient roosting space is available, flying‐foxes shift their roosting areas, which lessens their damage to vegetation over time	SEQ Catchments (2012), EcoLogical (2014)	Pallin (2000), Hall (2002)			

Note

Note that additional evidence from our 13‐month empirical study only addresses questions that require less than one year of data (i.e., intra‐annual patterns in roost structure). Statements not addressed with our empirical data are colored gray. An extended version of this table with details on study results is provided in Appendix S3.

Below, and in Table 2, we synthesize how commonly held understandings compare with existing literature and new data from our study.

### 3.1 Use of area

**“Some** **areas of permanent camps are more consistently occupied ('core areas’) than others”**

**FIGURE 2:**
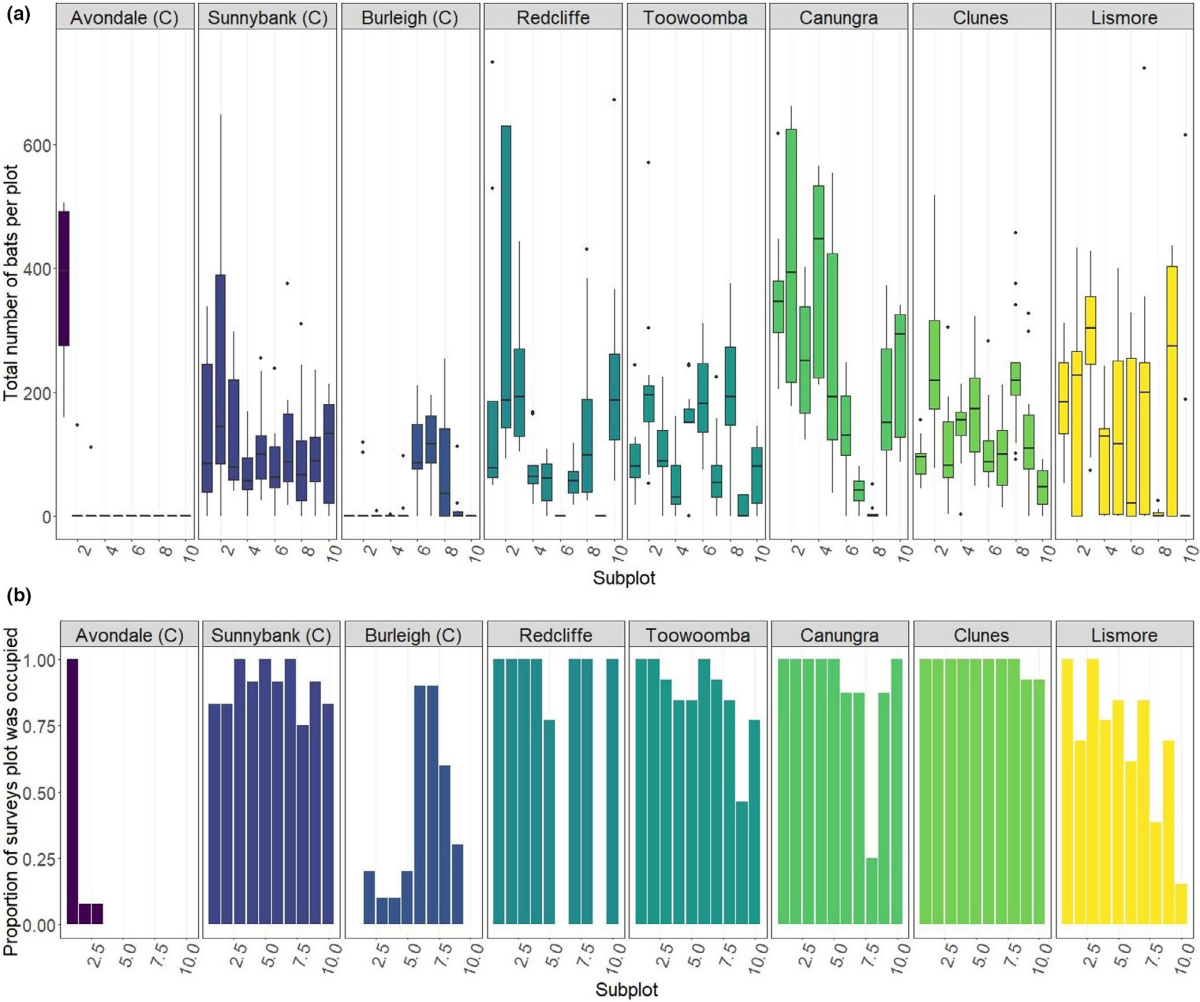
Occupancy of subplots across survey period, for surveys when at least one bat was present. (a) shows the total number of bats per subplot, and (b) shows the proportion of surveys the subplot was occupied. Facets/color indicates separate roost sites. “(C)” indicates roosts that have features of contemporary roost types (see Table 1). Note that construction works at the “Avondale” roost during the survey caused the bats to shift their roosting location, such that only one subplot was utilized thereafter

This understanding was widely reported in previous studies, with none contradicting it (Table 2). Consistent with these other studies (e.g., Nelson, 2020, 1965b; Welbergen, 2005), we observed some areas of roosts to be more consistently occupied than others (Figure 2). Occupancy of subplots ranged between 100% (30 subplots) and under 10% (15 subplots) across surveys when bats were present in roosts.

**“‘Core** **areas’ are more densely occupied than ‘peripheral areas’”**

**FIGURE 3:**
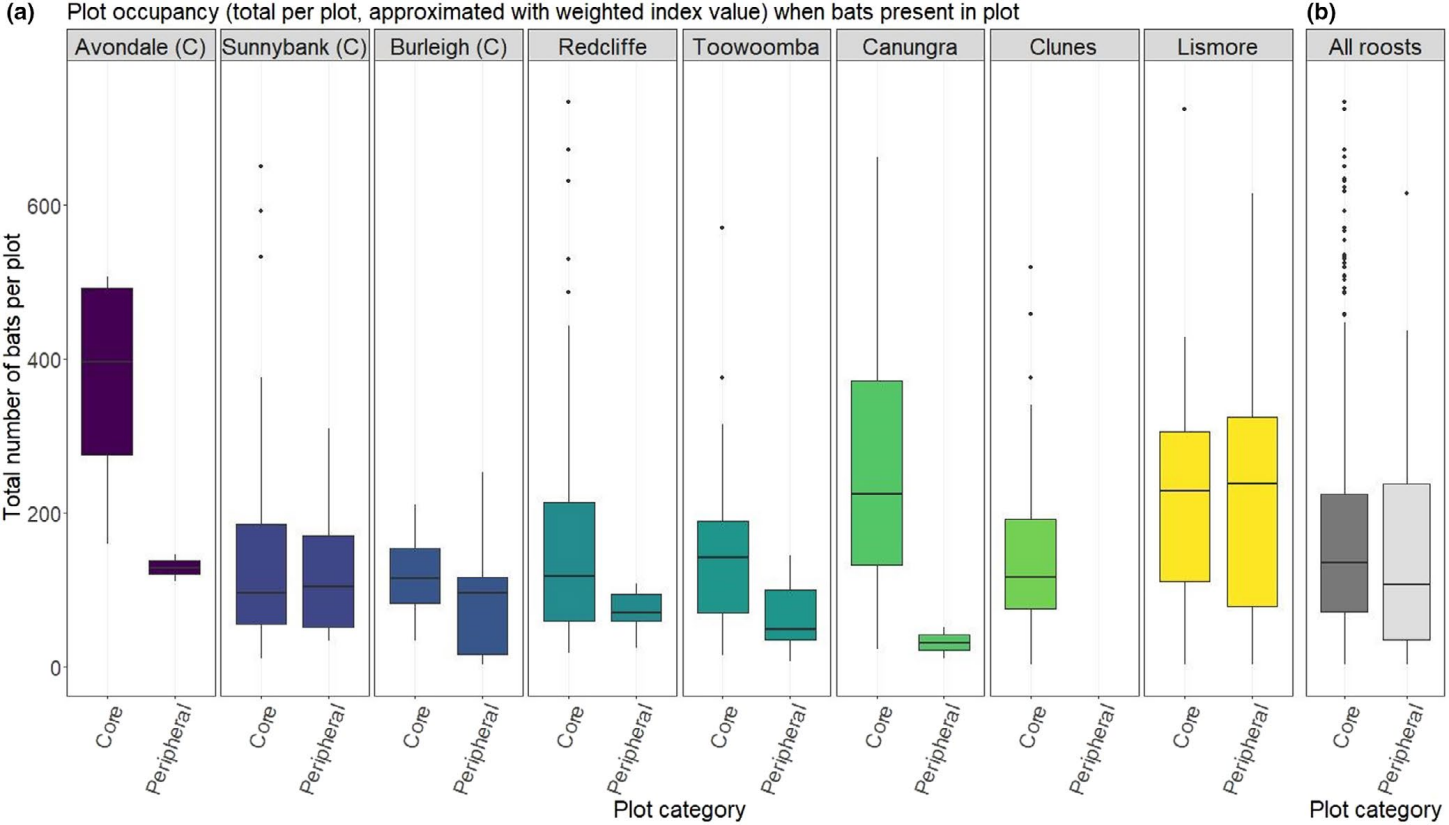
Occupancy of subplots in “core” and “peripheral” areas, shown by average total number of bats per occupied subplot across the survey period. Data are filtered to show numbers of bats when subplots were occupied (i.e., unoccupied subplots are removed). “Core” subplots were identified as those occupied in at least 80% of surveys (when bats present at the roost), and “peripheral” subplots as those occupied less than 80% of the time. (a) Shows areas split by roost site (facet and color), and (b) shows all roosts combined. Area displayed in subplot has been cropped to remove extreme outliers. “(C)” indicates roosts that have features of contemporary roost types (see Table 1)

Existing empirical data broadly supported this statement (Table 2). In our study, peripheral areas (those occupied less than 80% of the time) generally were less densely occupied than core areas, though density varied substantially across roost site, subplot, and session (all contributed substantially as random effects). Here, lower density refers to both a lower number of bats per subplot in peripheral subplots (−0.581 ± 0.177, *p* = .001, Figure 3), and a lower proportion of occupied trees (−0.222 ± 0.078, *p* = .005, Appendix S3). Within subplots, we also note that some trees were more consistently used than others, including trees that were occupied in 100% of surveys where bats were present at the roost (Appendix S1). The number of bats per tree in irregularly occupied trees (occupied <80% of the time) was typically lower than in regularly occupied trees (−0.606 ± 0.034, *p* < .001).

**FIGURE 4:**
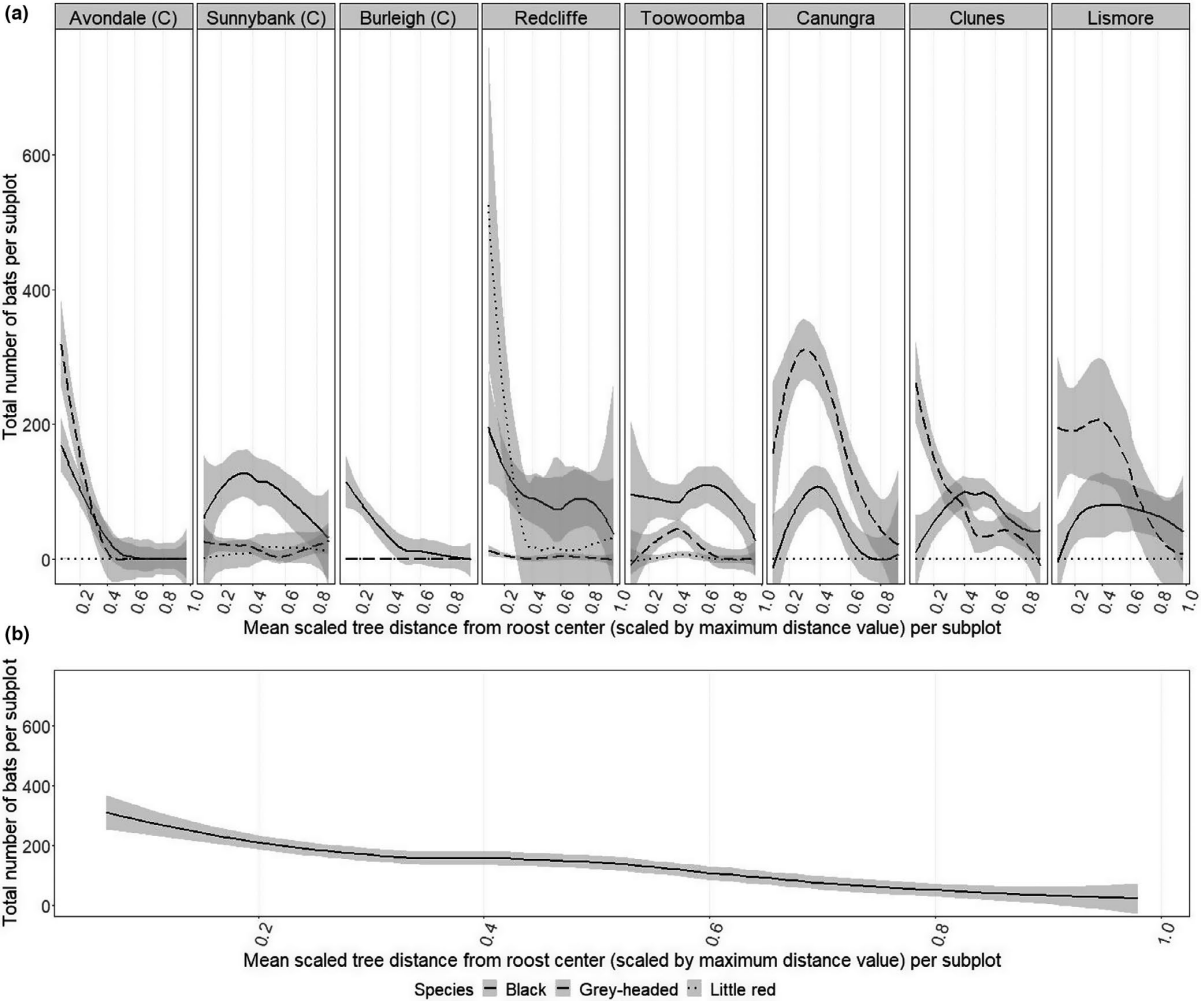
Distance from roost center and occupancy of bats, shown by the average total number of bats per occupied subplot during the survey period. Data are filtered to show numbers of bats when trees are occupied (i.e., unoccupied subplots are removed). Roost center is calculated for each survey as the centroid of the roost area at the time of the survey. Distance from the center is calculated as the mean distance of trees in each subplot from this centroid, scaled by the maximum observed distance value per session. (a) shows values per species (line type) split by roost (facets), and (b) shows species and roost combined. Trend line is by loess fit (local polynomial regression fit) with standard error bands (gray shading). “(C)” indicates roosts that have features of contemporary roost types (see Table 1)

We observed negative relationships between bat occupation metrics and distance from the roost center, including the number of bats per occupied subplot (−1.639 ± 0.016, *p* < .001, Figure 4) and proportion of occupied trees per subplot (−0.315 ± 0.034, *p* < .001, Appendix S3). This decline with distance from the center of subplot was largely driven by little red flying‐foxes (Figure 4). Roost site, subplot, and session also all contributed substantially as random effects (Appendix S3).

**“Roost** **area fluctuates with total abundance”**

**FIGURE 5:**
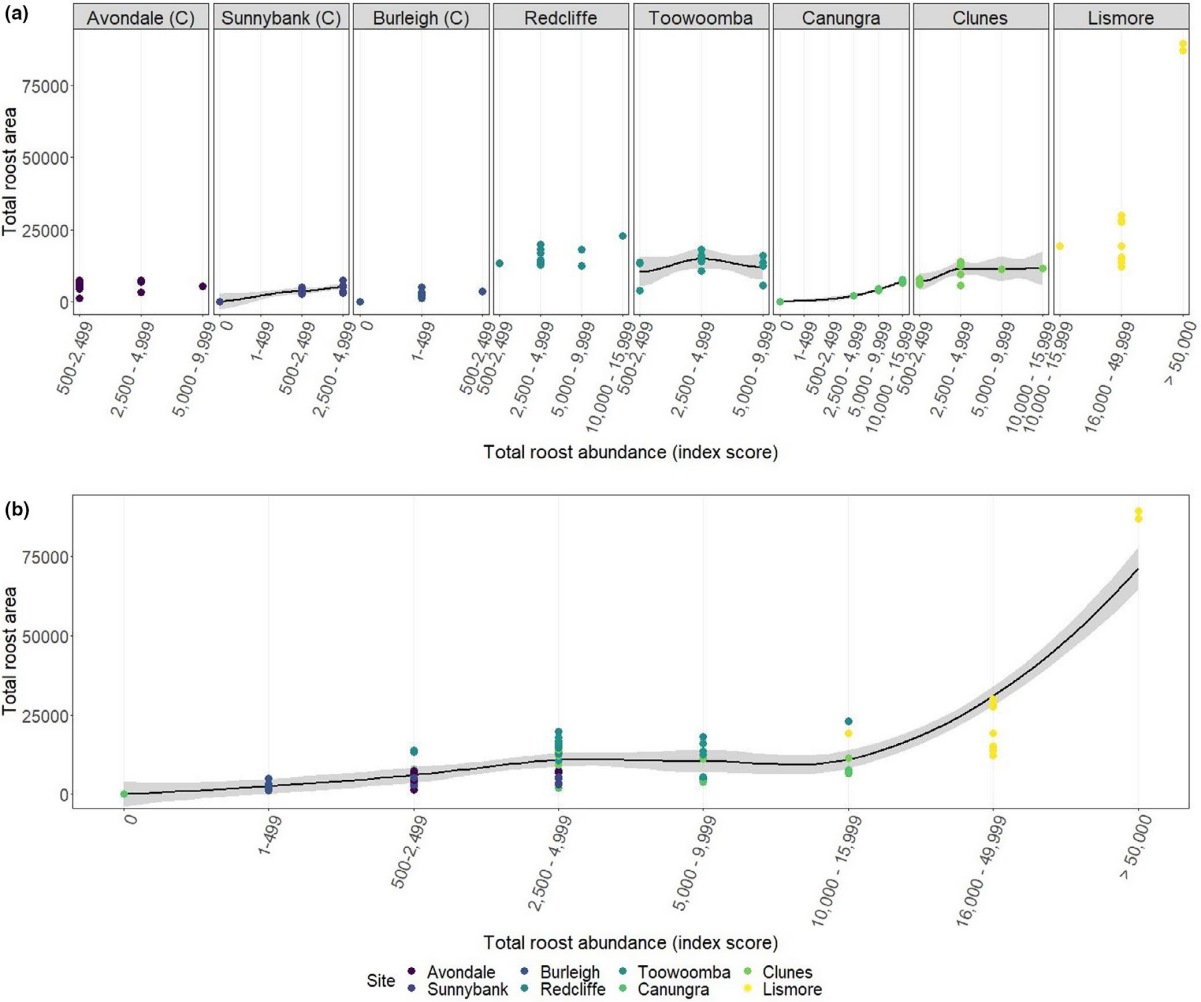
Relationship between total roost abundance (*x*‐axis) and total roost area (*y*‐axis) for each roost site. (a) shows relationship split by roost (facets), and (b) shows relationship with roosts combined. Trend line is by loess fit (local polynomial regression fit) with standard error bands (gray shading). Note that trend lines could not be fitted for all sites and are omitted. “(C)” indicates roosts that have features of contemporary roost types (see Table 1)

Studies have previously reported changes to total roosting area, but none to date have formally quantified the relationship between area and total bat abundance (Table 2). From our data, we observed substantial fluctuations in total roost area within some roost sites across monthly surveys, and overall, a positive relationship with total bat abundance. The extent of variation was variable across roosts, however (Figure 5). We note that relationships between total bat abundance and area were likely masked in many roosts by the large span of population values in some index categories (e.g., index six spans 16,000–49,999 bats). It is probable that data of finer resolution may have detected this relationship more strongly for roosts in this size range, but are not available in this dataset.

### 3.2 Spatial segregation of species

Results from our new dataset included systematic recording of the three species that occur in southeast Queensland—*P. alecto*, *P. poliocephalus*, and *P. scapulatus*. The majority of observations were made of *P. alecto* and *P. poliocephalus*, which occupy this region continuously through the year. *P. scapulatus* was found irregularly at some roosts, which is consistent with the seasonal migration patterns of this species (Nelson, 2017, 1965a). Fine‐scale spatial overlap between species was evaluated during surveys when multiple species were present (*N* = 73, 70.2% of surveys). Black and grey‐headed flying‐foxes co‐occurred in 65 surveys (62.5%), black and little red flying‐foxes co‐occurred in 17 surveys (16.3%), and grey‐headed and little red flying‐foxes co‐occurred in nine surveys (8.7%). We observed roost‐dependent support for spatial segregation of species.

**“Species** **share roosts sites, but segregate spatially within”**

**FIGURE 6:**
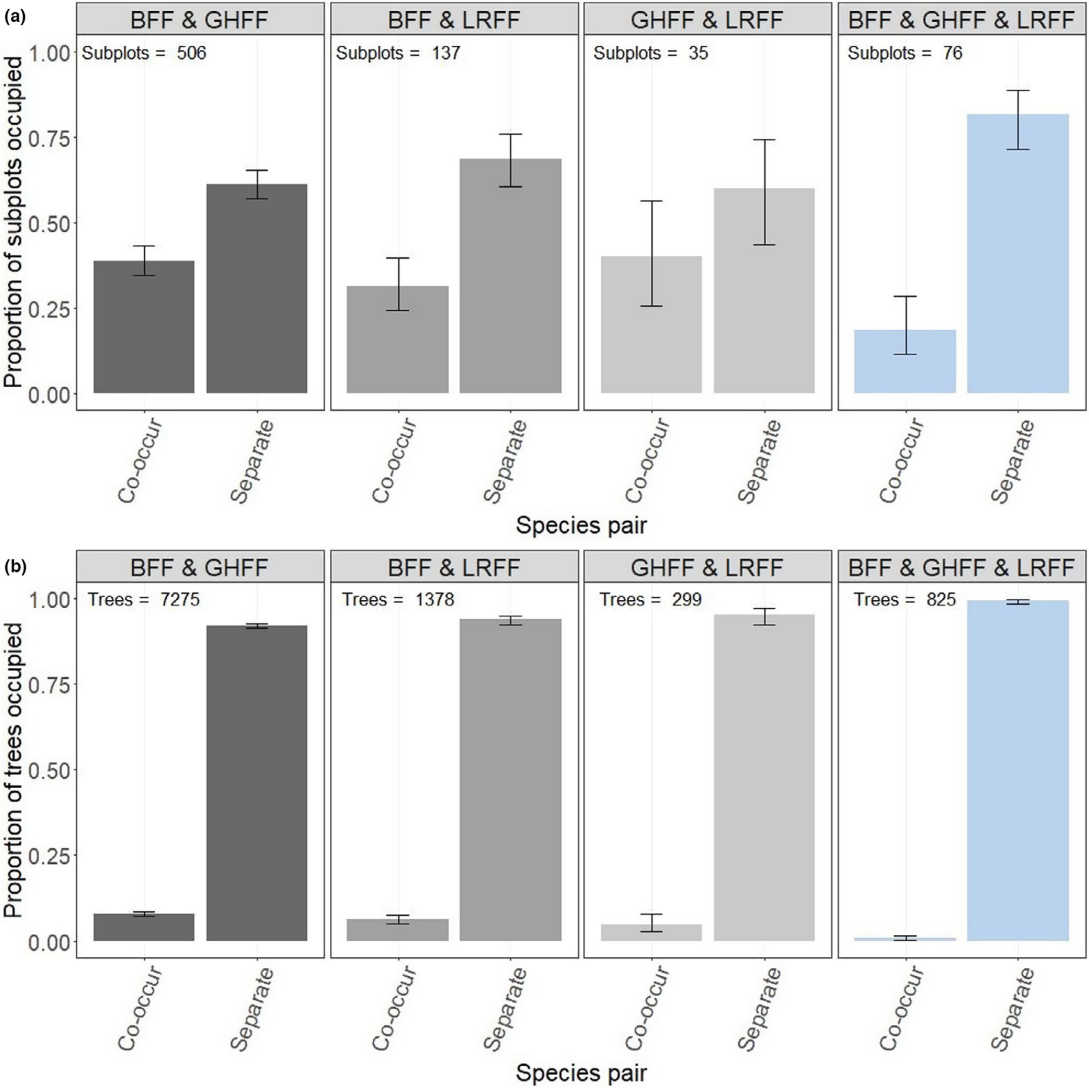
Co‐occupation of subplots (a) and individual trees (b) by species. Total subplots/total trees observed are shown in text labels and include subplots/trees across sessions where every bat species in the species comparison were present (e.g., for the black and grey‐headed flying‐fox comparison, only sessions where both black and grey‐headed flying‐fox were present were included in the subplot/tree tally). “BFF” refers to black flying‐fox, “GHFF” grey‐headed flying‐fox, and “LRFF” little red flying‐fox. Confidence intervals are binomial, calculated with a Wilson test

Observations from previous studies commonly report co‐occupation of roosts by multiple species, with anecdotal observations of inconsistent overlap or separation within and between trees (Table 2). We observed some horizontal spatial segregation of species, with species showing preference for discrete areas in roosts. In the “Lismore” roost, for example, black flying‐foxes were commonly distributed toward the eastern part of the roost and grey‐headed flying‐foxes in the western part of the roost (Appendix S4). Likewise, in the “Clunes” roost, black flying‐foxes were commonly observed toward the northeastern part of the roost and grey‐headed flying‐foxes in the southwestern part of the roost (Appendix S4). Of 659 occupied subplots across the survey period, only 34.1% (225, binomial confidence interval: 0.31–0.38) showed co‐occupation by two different species (Figure 6a). Co‐occupation of individual trees by two different species was also relatively low—across surveys where two species were present, 4.6%‐7.9% of occupied trees were co‐occupied by two species, versus 92.1%–95.4% that were occupied by only one species (Figure 6b). Only six trees were ever observed to occupy all three species at once.

**“Large** **influxes of species into roosts (especially little red flying‐foxes) can displace other species”**

Only one previous study had reported displacement by species, reporting an anecdotal observation of black and grey‐headed flying‐foxes being displaced by little red flying‐foxes (Table 2). Our quantitative data document changing distribution of regular species occupants in response to (“invading”) irregular species occupants, supporting this prior observation. Little red flying‐foxes, in particular, were observed to displace black and grey‐headed flying‐foxes from their usual roosting locations (most notably at the “Redcliffe” roost: Appendix S4). Black and grey‐headed flying‐foxes tended to co‐occur in roosts without too much impact on each other (Appendix S4).

**“Species** **roost at different heights”**

**FIGURE 7:**
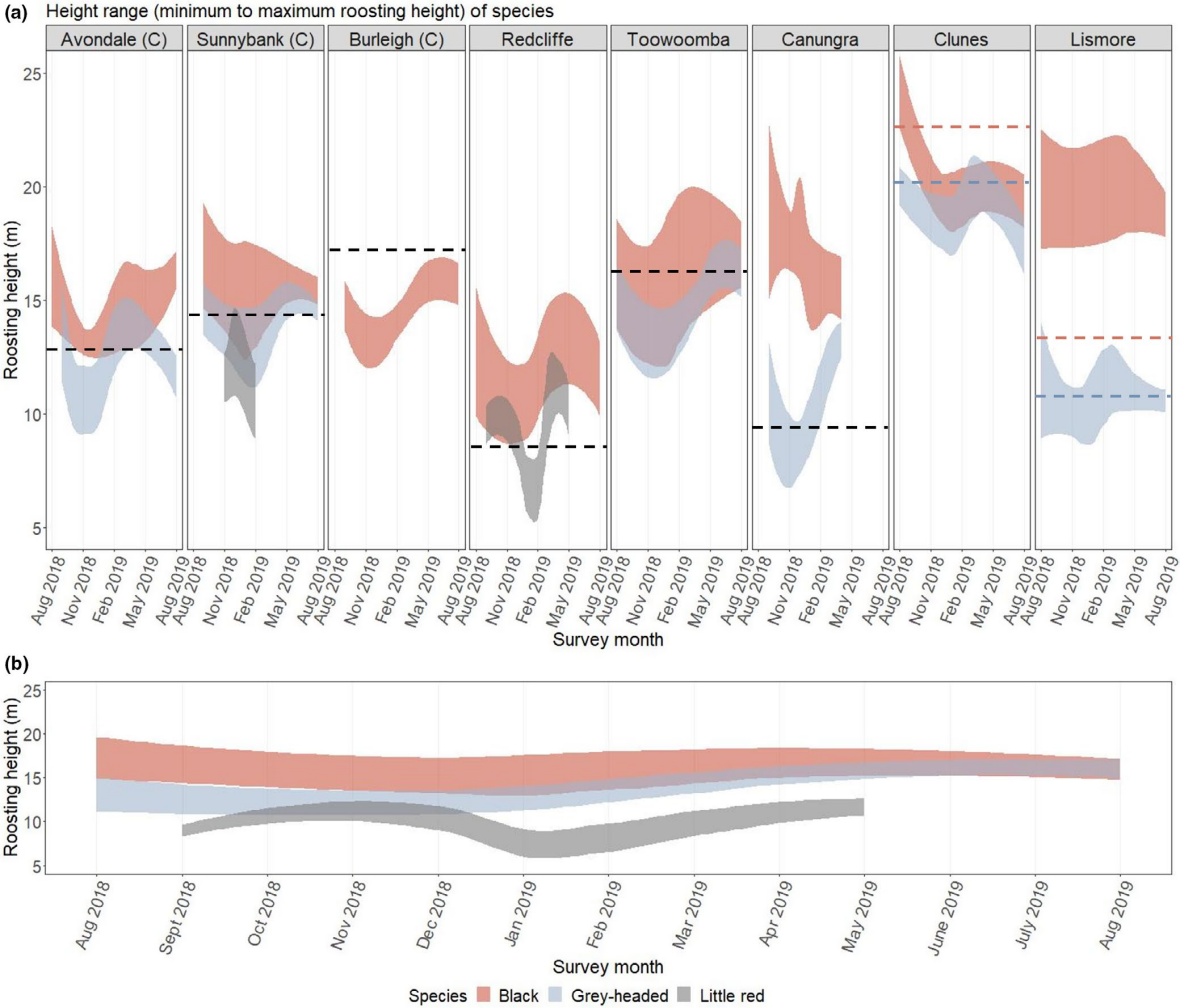
Difference in roosting height per species, over time. Fill shows average roosting height range per species (minimum height to maximum height). Fill boundaries (minimum and maximum curves) are by loess fit (local polynomial regression fit). (a) shows relationship split by roost (facets), and (b) shows relationship with roosts combined. In (a), dashed line represents the average canopy height per site; for roost sites where species occupy distinctly different areas (“Clunes” and ‘Lismore”), canopy height is split by areas the species predominantly occupy. “(C)” indicates roosts that have features of contemporary roost types (see Table 1). Note that height data are taken from the tree subset only (up to *N* = 60 per roost site) and that trend lines could not be fitted for all site‐by‐species combinations and are omitted

Previously, only one study had formally documented differences in roosting height between species (Table 2). This included a record of black flying‐foxes and grey‐headed flying‐foxes only and did not provide measures of absolute height (rather, roosting in different quadrants of trees) (Welbergen, 2005). From our new dataset, we observed segregation of species by roosting height, with black flying‐foxes typically showing the highest roosting heights (average maximum height with interquartile range: 18.0, 14.6–21.0; average minimum height with interquartile range: 14.3, 11.3–17.2), followed by grey‐headed (maximum: 15.1, 11.2–18.9; minimum: 12.6, 8.8–16.2), then little red flying‐foxes (when present) (maximum: 11.4, 9.2–13.6; minimum: 8.8, 7.1–10.4) (Figure 7). Note, however, that topographical variation (change in ground height, e.g., from creeks and small crests) within roosts was not taken into consideration in measures of height. Differences in heights presented here reflect a relative difference in roosting heights from the ground within trees, but may not reflect true, realized height relative to the canopy.

### 3.3 Demographic/social structure

**“The** **majority of roost trees are occupied by mixed groups of adults, with territories comprised of a single male and one or more females and their dependent young”**

We commonly observed roost trees to be occupied by mixed groups of sexes, with a single tree occupied by one or more males, and one or more females and their dependent young. This is inconsistent with general knowledge based on historical studies such as Nelson (2017, 1965a) and Nelson (2020, 1965b), but consistent with more contemporary observations (Table 2). We also observed cases where trees were occupied by entirely male individuals (consistent with reports of “bachelor male” trees in Markus (2002)). We would note here that a single tree may contain multiple male territories (Connell, 2003; Markus, 2002), and the survey methods did not allow inference on the composition of individual territories, only individual trees. The proportion of males per tree appeared to follow seasonal patterns that were mostly consistent between black and grey‐headed flying‐foxes within roosts (Appendix S3). Some roosts (“Toowoomba,” “Avondale,” “Lismore”) showed an increase in the proportion of males per tree after parturition in September/October, while other roosts (“Sunnybank,” “Canungra”) decreased immediately after this time. We also did not observe complete segregation of sexes at any time of the year, in contrast to Nelson (2020, 1965b) who noted complete segregation between September until early December, and March to April.

Interestingly, we observed an overall female bias to roosts (1:0.76 female:male ratio, averaged across roost sites and sessions), which held across grey‐headed (1:0.64) and black flying‐foxes (1:0.92) but not little red flying‐foxes, which had a male bias (1:1.60 female:male ratio). When split by roost type (contemporary/noncontemporary; Table 1), contemporary roosts consistently showed a female bias (black flying‐fox 1:0.74, grey‐headed flying‐fox 1:0.45, and little red flying‐fox 1 0.39), while noncontemporary roosts either flipped to a male bias (black flying‐fox 1:1:02 and little red flying‐fox 1:1.90) or showed an increase in male occupation (grey‐headed flying‐fox 1:0.69). The same pattern held when comparing urban (0 km from urban edge) and peri‐urban (>10 km from urban edge; see Table 1) roosts (black flying‐fox: urban 1:0.77 versus peri‐urban 1:1.26, grey‐headed flying‐fox: urban 1:0.54 versus peri‐urban 1:0.65). The average proportion of females with young one–three months after parturition (indicative of effective population size) was reasonably high, with 1:0.44 females:females with young observed in both black and grey‐headed flying‐foxes, averaged over the period October–December. The ratio of females:females with young was similar between contemporary and noncontemporary roosts (black flying‐fox: contemporary 1:0.51 versus noncontemporary 1:0.40, grey‐headed flying‐fox: contemporary 1:0.47 versus noncontemporary 1:0.43) and urban and peri‐urban roosts (black flying‐fox: urban 1:0.51 versus peri‐urban 1:0.33, grey‐headed flying‐fox: urban 1:0.41 versus peri‐urban 1:0.46). We did not observe any little red flying‐foxes with young.

**“Dominant individuals (defined as reproducing males and females) occupy the center of roosts and subdominant individuals (defined as nonreproducing males and females) the outer area”**

**FIGURE 8:**
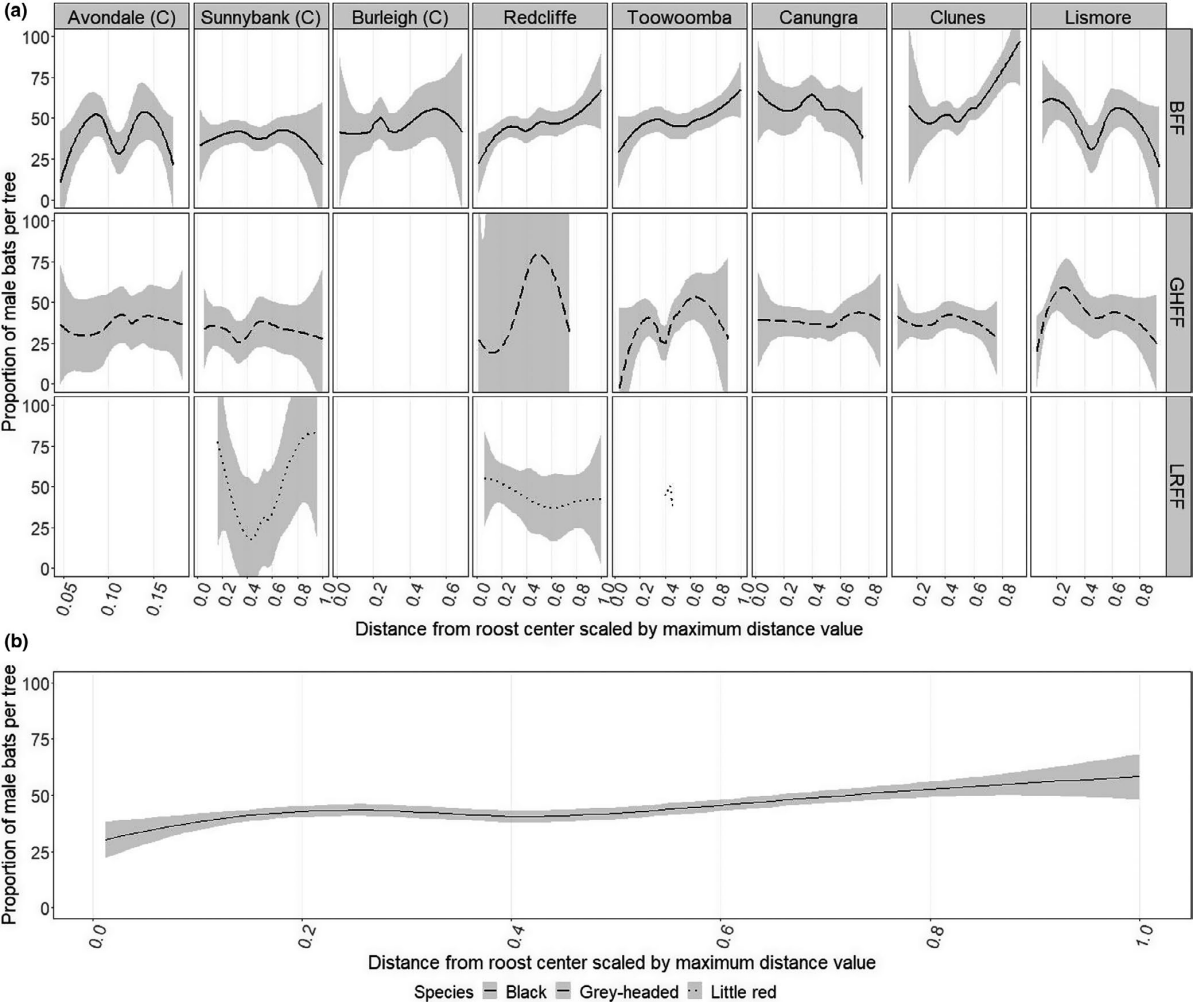
Proportion of male bats per occupied tree versus distance of tree from the roost center, scaled by the maximum distance value observed per session. (a) shows values per species (row facet) split by roost (column facet); (b) shows combined species value pooled by roost. Trend line is by loess fit (local polynomial regression fit) with standard error bands (gray shading). “BFF” refers to black flying‐fox, “GHFF” grey‐headed flying‐fox, and “LRFF” little red flying‐fox. “(C)” indicates roosts that have features of contemporary roost types (see Table 1)

From our new dataset, we observed that the proportion of males per tree increased with distance from the roost center (0.15 ± 0.039, *p* < .001), though this effect was relatively small and variable across roosts and species (Figure 8). We assume that only dominant reproducing males share their territory with females and their young and use the proportion of males per tree as a proxy for dominance structure in the roost. A lower proportion of males in trees closer to the center of roosts may indicate that dominant individuals occupy the center of some roosts and subdominant individuals the outer area. The small effect sizes observed would suggest that there is no clear spatial structure to reproductive groupings or dominance groupings, however. This can be seen also in maps showing male composition by tree relative to the roost perimeter, given in Appendix S5. Prior studies have reported inconsistent spatial patterns in flying‐fox occupation (Table 2).

### 3.4 Roost abundance/occupancy

**“Individual roosts have distinguishable seasonal patterns of abundance and occupation,” “Intra‐ and interannual variations in abundance can be extreme,” and “Roost abundance peaks in March”**

**FIGURE 9:**
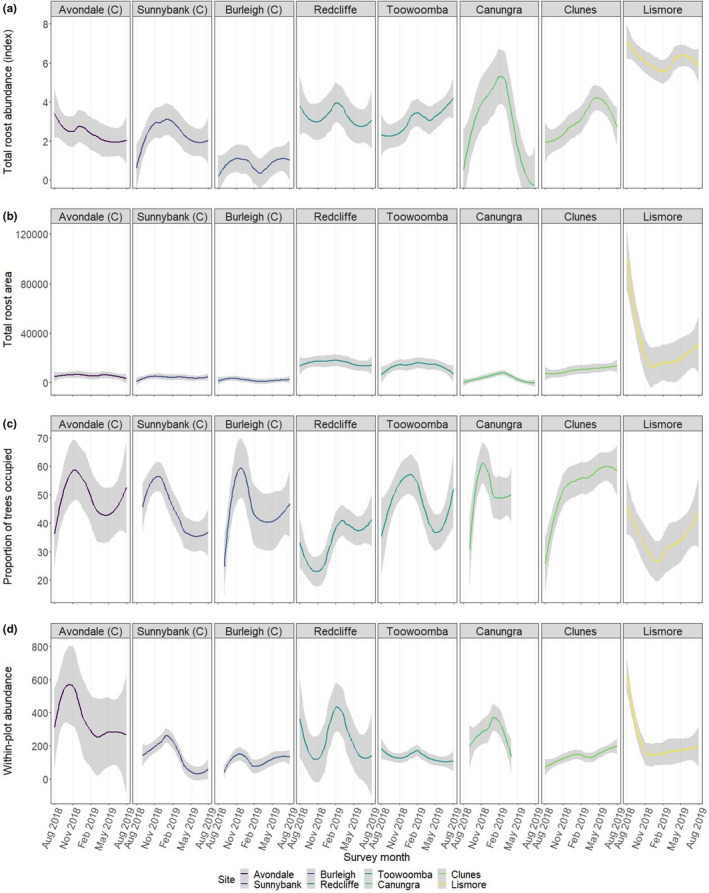
Different scales of bat abundance measures through time. (a) shows total roost abundance; (b) shows total roost area; (c) shows the proportion of occupied trees per subplot; and (d) shows the total abundance of occupied subplots. Total roost abundance is measured by an index score of abundance: 1 = 1–499 bats; 2 = 500–2,499 bats; 3 = 2,500–4,999 bats; 4 = 5,000–9,999 bats; 5 = 10,000–15,999 bats; 6 = 16,000–49,999 bats; and 7 = >50,000 bats

Prior studies reported inconsistent patterns in occupancy and abundance (Table 2). In our dataset, seasonal patterns in abundance and density were roost‐specific (Figure 9). Some roosts showed patterns consistent with the general notion that total roost abundance peaks toward March (Nelson, 2020, 1965b; State of NSW & Office of Environment & Heritage, 2018) (e.g., “Redcliffe,” “Canungra” and “Clunes”). Others showed no considerable fluctuation in abundance (“Burleigh”) or peaks at other times (“Toowoomba,” “Sunnybank,” “Avondale,” “Lismore”) (Figure 9). The latter cases potentially highlight that population dynamics are more strongly driven by local dynamics in these roosts (e.g., food availability) (Eby et al., 1999; Giles et al., 2016; Parry‐Jones & Augee, 1992; Parry‐Jones & Augee, 2001), than reproductive cycles as described in Nelson (2020, 1965b). Little red flying‐foxes showed seasonal trends in occupancy and density, peaking in February–March, reflecting their summer influx into coastal eastern Australia to feed on blossom (Ratcliffe, 1931; Sinclair et al., 1996) (Appendix S3). Seasonal trends in grey‐headed and black flying‐fox numbers were less consistent between roost sites (Appendix S3).

## 4 DISCUSSION

The success of efforts to conserve Pteropodid bats across their distribution relies on effective population and habitat management. Pivotal to this is a baseline understanding of species ecology and behavior, which is currently lacking for the majority of these species (Fujita & Tuttle, 1991; Mickleburgh et al., 2002). Here, we provide a synthesis of all existing literature, as well as an unprecedented empirical dataset, to meet that need for Australian species of *Pteropus*. We highlight that many existing beliefs on which conservation and management decisions are based are unsupported or outdated, and suggest that management plans should be updated to incorporate improved knowledge. Most importantly, we highlight that a one‐size‐fits‐all approach to roost management will be inappropriate, given the extent of variation between sites even within a regional area. Roost management guidelines need to be changed to promote a more tailored approach that requires preliminary data acquisition before management plans are formulated and approved.

### 4.1 Existing understanding not supported

**“Individual** **roosts have distinguishable seasonal patterns of abundance and occupation”**

All roost sites in our empirical dataset were occupied continuously throughout the year by both sexes. This type of roost occupation has been noted from 1981 onward (Parry‐Jones, 1985; Puddicombe, 1981) and has become common in recent decades (e.g., Aston, 1987; Eby, 1991; Larsen et al., 2002; Van der Ree et al., 2006). This pattern of occupation contrasts to the “summer” and “winter” pattern described historically by Nelson (2017, 1965a) and Nelson (2020, 1965b) and cited in the *Flying‐fox Roost Management Guideline* for Queensland, where “summer roosts” of reproducing individuals would form between ~September/October and April/May, and “winter roosts” of dispersed animals would form between April/May and September (Nelson, 2017, 1965a, 2020, 1965b; Parry‐Jones & Augee, 1991, 1992; Ratcliffe, 1931). For these roost types, overwintering animals at summer roosts were rare and, when present, were documented as being predominantly juveniles or lone adult males (Nelson, 2020, 1965b).

While seasonally occupied colonies are still observed (e.g., Klose et al., 2009), an increasing number of roosts are now consistently occupied year around, particularly in urban areas (Parry‐Jones & Augee, 2001; Tait et al., 2014). The cyclic patterns of summer aggregation and winter dispersal were originally thought to reflect social drivers and availability of resources (Parry‐Jones & Augee, 1992). Specifically, territory formation (from January) and conception (from ~March) (*P. poliocephalus* and *P. alecto*) (Welbergen, 2005) coupled with abundant flowering of native flora in these months (Nelson, 2017, 1965a) were understood to drive and support aggregative living in summer/autumn, while decreased food availability and the cessation of mating from ~May triggered the animals to disperse and adopt a less‐gregarious living style in winter (Parry‐Jones & Augee, 1992). This ecology has changed in more recent decades, where continuous availability of exotic foods in urban areas has reduced the need for migratory behaviors and allows aggregate groups to remain year‐round (Parry‐Jones & Augee, 2001; Williams et al., 2006).

Policy documents containing only historical information on flying‐fox occupation patterns (including the most recent *Flying‐fox Roost Management Guideline* for Queensland: State of Queensland Department of Environment and Science (2020)) are of concern, as recommendations based on historical usage patterns may be inconsistent with current usage patterns, particularly in urban areas where occupation patterns have changed the most (Larsen et al., 2002; Tait et al., 2014), and where human–bat conflict is often the highest (Kung et al., 2015). Roost monitoring prior to management actions should encompass every season, and not assume that bats will disperse in winter. Similarly, contemporary overwintering roosts commonly contain individuals from all age and sex groups and may be consistently utilized through time (Larsen et al., 2002; Tait et al., 2014).

**“The majority of roost trees are occupied by mixed groups of adults, with territories comprised of a single male and one or more females and their dependent young,” and “Dominant individuals (defined as reproducing males and females) occupy the center of roosts and subdominant individuals (defined as nonreproducing males and females) the outer area”**

These historic perspectives also describe the complete separation of males and females between September until early December (the period immediately before parturition, during lactation, and before conception) and again post‐March (after conception) (Nelson, 2017, 1965a, 2020, 1965b). During these times, animals were historically noted to segregate by tree or height, such that all social contacts were between individuals of the same sex. However, these observations contrast with more recent observations of flying‐fox social groupings (Eby et al., 1999; McWilliam, 1984; Puddicombe, 1981; Welbergen, 2005), and observations from this study. In contemporary roosts, mixed‐sex groups are commonly present all year around, such that males and females co‐occur in the roost and within trees year around.

In addition, more recent observations, and results from this study, suggest that there is no clear spatial structure in the distribution of the sexes within the roost. This contrasts with the common perspective that dominant reproducing individuals—particularly reproducing females—occupy the center of roosts, and nondominant individuals—including weaned juveniles—occupy the edges of roosts (Table 2). Puddicombe (1981) notes that reproductive groups (mixed groups of males, females, and their young) were uniformly distributed through the camp and present in peripheral areas (McWilliam, 1984). Additionally, in this study we observed randomly distributed groups of mixed males and females, and groups of all‐male trees. While we did not systematically record age of bats, as estimating age from observations at a distance is not always possible, on few occasions we did observe what looked to be all‐juvenile trees. These observations of all‐juvenile trees were not at the edges of the roosts. This difference in sex and age structure of roosts potentially reflects the change in occupancy patterns in flying‐fox roosts, where aggregative living was historically believed to be driven by strong social drivers (i.e., mating), whereas aggregative living in contemporary roosts is thought to be driven by continuous resource availability in the urban environment (Parry‐Jones & Augee, 2001; Williams et al., 2006). The observations will have implications for current management plans. Specifically, in support of current guidelines, managers should avoid management actions during times of the year when females are in late stages of gestation and have dependent young that cannot fly on their own (as per Commonwealth of Australia, 2015; Department of Environment & Science, 2020a). Importantly (and in contrast to current guidelines), actions scheduled within this time should note that restricting work to edges of roosts will likely not circumvent disturbances to gestating females and dependent young.

A recent tracking study demonstrated a sex‐based difference in visitation to major urban areas, with males but not females suggested to roost more often in major urban areas (Meade et al., 2021). From this information, Meade et al. (2021) suggest there may be a male bias in urban roosts and that conservation efforts should be directed at nonurban roosts where the effective breeding population is likely to be higher. The present study observed an overall female bias in roosts (1:0.76 average female:male ratio), which held across both grey‐headed (1:0.64) and black flying‐foxes (1:0.92) but not little red flying‐foxes (1:1.60). In contrast to predictions by Meade et al. (2021), this female bias became more extreme in urban areas (1:0.54 for grey‐headed flying‐foxes, and 1:0.77 for black flying‐foxes). A greater representation of males was only observed in peri‐urban areas more than 10 km from the urban edge (1:0.65 for grey‐headed flying‐foxes, and 1:1.26 for black flying‐foxes). In addition, the ratio of total females to females with pups in the months after parturition (October–December) was similar between roosts in urban areas (1:0.41 for grey‐headed flying‐fox, and 1:0.51 for black flying‐fox) and roosts outside urban areas (1:0.46 for grey‐headed flying‐fox, and 1:0.33 for black flying‐fox), suggesting that both roost types have a high potential effective breeding population. We suggest both roosts types should receive adequate management and conservation attention.

### 4.2 Existing understanding supported, but conditional on roost site and local conditions

**“Roost** **abundance peaks in March” and “Intra‐ and interannual variations in abundance can be extreme”**

March was identified in some management documents as being the time for peak abundance in flying‐fox roosts (e.g., State of Queensland Department of Environment & Science, 2020). However, studies on *P. poliocephalus and P. alecto* identify a typical pattern of increasing abundance from September–October (when females give birth) until a peak in January–February (when the season's young are able to fly independently) (Eby, 1991; Eby & Palmer, 1991; Nelson, 2020, 1965b; Parry‐Jones & Augee, 2001). Roost sizes then decrease during March–April (the period of mating) to low winter counts in continuously occupied/overwintering roosts, or zero winter counts in seasonally occupied summer roosts (Eby, 1991; Eby & Palmer, 1991; Nelson, 2020, 1965b). These studies note that cyclical patterns of occupation are driven by reproductive factors (i.e., timing of birth and independent flight), but highlight that irregular, local dynamics of food availability can superimpose variability into these patterns of abundance (Parry‐Jones & Augee, 1992). Indeed, many studies note high intra‐ and interannual variability in abundance. Parry‐Jones and Augee (2001), for example, note that animals from their study roost appeared to migrate away and decrease in abundance in response to a blossoming event, presumably to move to a roost in closer proximity to the blossoming.

In our study, some roosts showed patterns consistent with a total roost abundance peak toward March (e.g., “Redcliffe,” “Canungra,” and “Clunes”). Others showed either no considerable fluctuation in abundance (“Burleigh’) or peaks at other times (“Toowoomba,” “Sunnybank,” “Avondale,” “Lismore”). Drivers of peaks were variable between roosts. For the “Redcliffe” roost, seasonal migration of little red flying‐foxes from ~January 2019 contributed to a peak in abundance around March (see species abundance plots in Appendix S3). For the “Lismore” roost, a blossoming event in winter 2018 triggered an influx of nomadic bats into the population, driving the peak observed in August 2018. Dynamics observed in other roosts were likely the result of local dynamics of food availability.

We note also that estimates of abundance from our study were much smaller than those of historical estimates. Ratcliffe (1931) describes “small” roosts as ~5,000–10,000 animals, “medium” as 10,000–50,000, and “large” as anything over this size. Ratcliffe (1931) also report roosts in northern Queensland with bats “into the millions” (Red River) and “exceeded a quarter of a million, possibly considerably” (Burnett River). Likewise, Lunney and Moon (1997) report historical observations of flying‐foxes in the Richmond Valley (1870s) as into the millions. The maximum roost site observed in this current study was ~95,000, recorded at the Lismore roost in August 2018 in response to a local eucalyptus flowering event. Roost sizes of <5,000 were more common for the roost sites surveyed and, extending from the sizes in Ratcliffe (1931), may constitute a new category of “very small.” Local management areas should expect that local conditions can change substantially and rapidly for flying‐fox populations, resulting in population changes outside of times predicted by demographic driven dynamics alone. An understanding of the timing and productivity of flower resources within the feeding range of roosts is likely to be of greater importance to forecasting and interpreting large population fluctuations than are reproductive considerations.

### 4.3 Existing understanding supported

**“Some areas of permanent camps are more consistently occupied ('core areas’) than others,” “‘Core areas’ are more densely occupied than ‘peripheral areas’,” and “Roost area fluctuates with total abundance”**

Variability in the usage and occupation of areas within roosts has been highlighted in management documents (e.g., SEQ Catchments, 2012). This includes more persistent usage of “core” areas, higher occupation of “core” areas, and variability in the roost perimeter (reflecting expansion and contraction from the core area). All existing literature (to our knowledge) and the new data from our study support these understandings. We would note, however, the distinction between a “core/peripheral” roost area and a “central/edge” roost area. We defined the core area based on consistency of occupation, not spatial location. Areas identified to be “core” were not necessarily in the center of the roost (see location of roost centroid relative to the roost perimeter and surveyed subplots, in Appendix S4). This distinction has not necessarily been made in literature and management plans to date but has important implications for the interpretation of “core” roosting areas, and management recommendations specific for “core/central” or “peripheral/edge” areas. For example, it cannot be assumed that buffer creation via vegetation removal from the roost edge will not affect a “core” area of bat roosting, and so will not have a substantial impact on flying‐foxes. Management activities should be prescribed for specific zones in roosts, based on prior monitoring of the roost, and recognizing the ecological importance of different areas (Ku‐ring‐gai Council, 2018; Pallin, 2000). In addition, prior monitoring of core/peripheral roosting areas will be important to inform the location and potential effectiveness of buffer creation. Given the potential for roost area to fluctuate with abundance, creation of buffers via vegetation removal may reduce the area of normal roost habitat available, and result in an expansion into new areas when flying‐fox numbers increase (as noted in Currey et al., 2018). The prescription of buffers should be planned with care to avoid unintended outcomes during periods of high population abundance.

**“Species share roost sites, but segregate spatially within,” “Large influxes of species into roosts (especially little red flying‐foxes) can displace other species,” and “Species roost at different heights”**

The range of black flying‐foxes underwent a phase of rapid southern expansion in the late 1990s and early 2000s, increasing the area of overlap with grey‐headed flying‐foxes (Roberts et al., 2012a). As the two species co‐occupy roosts where their distributions overlap, this process has substantially increased the number of roosts occupied by both species, and thereby increased the pertinence of understanding the structure of mixed‐species roosts. There has been relatively little formal documentation of species overlap and segregation within roosts. Ratcliffe (1932) noted that sections of roosts were occupied by different species—specifically, that little red flying‐foxes and black flying‐foxes occupied different areas. Some horizontal separation has also been noted by Nelson (2020, 1965b) and Klose et al. (2009), and notes of displacement by little red flying‐fox have been described in Birt and Markus (1999). We contribute quantitative, spatial information on the extent and overlap of little red flying‐fox, black flying‐fox and grey‐headed flying‐fox roosting, extending on the predominantly anecdotal observations underlying management documents to date. Findings from our data support common understandings of flying‐fox roost structure: species commonly showed preferences for discrete areas of roosts, and even more commonly, preference for occupation of separate trees. We also observed segregation of species by roosting height, with black flying‐fox foxes showing the highest roosting, followed by grey‐headed flying‐foxes and little red flying‐foxes. These findings flag the importance of species monitoring of roost sites prior to management interventions. It cannot be assumed, for example, that species occupy areas of the roost uniformly, and management actions need to consider areas that may be more or less important to vulnerable species, such as the grey‐headed flying‐fox. These results also give interesting insights into understanding disease transmission dynamics within roosts, relating to the extent of mixing of primary host species (e.g., black flying‐foxes for Hendra virus) and other species presumed to be incidental hosts (e.g., grey‐headed and little red flying‐foxes).

### 4.4 Final comments and implications for roost management

State‐level management guidelines, including the *Flying‐fox Camp Management Policy* (State of NSW & Office of Environment & Heritage, 2018) and the *Flying‐fox Roost Management Guideline* (State of Queensland Department of Environment & Science, 2020), outline several camp‐based management approaches that involve the modification or removal of vegetation within roost sites. “Routine camp management actions” include the removal of tree branches or whole trees, weed removal, trimming of understory vegetation, and minor habitat augmentation. The aims of such actions are often to encourage roosting in alternative areas of the roost (e.g., EcoLogical, 2014; Geolink, 2010) or to increase the sustainability of existing roosting habitat for flying‐foxes (e.g., Ku‐ring‐gai Council, 2018). These actions are considered to be low impact activities (Department of Environment & Science, 2020b) and do not require referral under the EPCB act (Commonwealth of Australia, 2015); however, these actions may considerably alter the structure of roost vegetation and decrease the suitability of a roost as habitat (Ku‐ring‐gai Council, 2018). For example, the removal of mature weed vines in the canopy and midstory, as well as the clearing of understory, can reduce the structural complexity of roost vegetation. This may have immediate and direct effects on roosting flying‐foxes and may accidentally cause bats to disperse or adjust use of roost trees in ways contradictory to conflict management. This may also have long‐term, indirect implications for the ability of flying‐foxes to survive extreme weather events, by altering roost macroclimate and removing physical refuge needed at times of extreme heat (Welbergen et al., 2008).

Individual‐ and council‐level roost management plans developed by local governments under the guidance of these policies commonly utilize these vegetation management measures (e.g., EcoLogical, 2014; Ku‐ring‐gai Council, 2018; Logan City Council, 2015; Sunshine Coast Regional Council, 2016), though the long‐term implications for flying‐foxes of vegetation works are rarely noted (with the exception of Ku‐ring‐gai Council, 2018). We recommend that vegetation removal should not be considered low impact by default. Routine management actions should follow a mosaic pattern (State of NSW & Department of Planning Industry & Environment, 2019), or target weeding on a weed‐by‐weed case basis (Ku‐ring‐gai Council, 2018), and seek to maintain refuges in the mid‐ and lower storys at all times. Special care not to disturb bats should be taken in identified core areas of the roost.

## 5 CONCLUSION

This study takes a thorough, multifaceted approach to better understand the ecology of flying‐fox roost use and structure in Australia. We build upon broadscale knowledge of historic roosting occupancy and abundance patterns, and provide updated baseline information on roosting structure in urban and peri‐urban roosts by providing fine‐scale spatial, and temporal data on roost and tree use. Specifically, we demonstrate high variation in patterns of occupancy and abundance between roosts sites, and provide updated demographic information including the spatial and temporal distributions of males and females within roosts. We also show evidence of sympatry and indirect competition between species, including spatial segregation of black and grey‐headed flying‐foxes within roosts, and seasonal displacement of both species by little red flying‐foxes. The outcomes of this research will be of immediate, practical benefit to management and conservation of flying‐fox roosts in Australia, and meet research needs specifically identified in the draft Recovery Plan for the vulnerable grey‐headed flying‐fox. The level of spatial and temporal detail provided in our empirical study will be important in designing management plans that are sensitive to flying‐fox habitat needs, and in identifying and protecting important habitat areas within roosts that are reflective of current movements and preferences. Most importantly, we highlight that a one‐size‐fits‐all approach to roost management will be inappropriate, given the extent of variation between sites even within a regional area. Fine‐scale information on roost tree preferences will also improve understanding of the potential impacts of existing conflict management strategies involving vegetation removal, including buffer creation, and can guide vegetation removal efforts to heed these habitat requirements. This information is timely and much needed in advance of the recently announced Environmental Trust grants program for flying‐fox habitat restoration, and in the face of continued and increasing urbanization of flying‐foxes in Australia.

## CONFLICT OF INTEREST

The authors have no conflicts of interest to declare.

## AUTHOR CONTRIBUTION

**Tamika J. Lunn:** Conceptualization (lead); Data curation (lead); Formal analysis (lead); Funding acquisition (lead); Investigation (lead); Methodology (lead); Project administration (lead); Visualization (lead); Writing‐original draft (lead); Writing‐review & editing (equal). **Peggy Eby:** Conceptualization (supporting); Methodology (supporting); Supervision (equal); Writing‐review & editing (equal). **Remy Brooks:** Data curation (supporting); Investigation (supporting); Writing‐review & editing (supporting). **Hamish McCallum:** Formal analysis (supporting); Supervision (equal); Visualization (supporting); Writing‐review & editing (equal). **Raina K. Plowright:** Supervision (equal); Writing‐review & editing (equal). **Maureen K. Kessler:** Conceptualization (supporting); Writing‐review & editing (supporting). **Alison J. Peel:** Formal analysis (supporting); Supervision (equal); Visualization (supporting); Writing‐review & editing (equal).

## Supporting information

Appendix S1‐S5

## Data Availability

Summarized data are available on Dryad https://doi.org/10.5061/dryad.g4f4qrfqv. Annotated R code is available on GitHub at: https://github.com/TamikaLunn/FF‐roost‐ecology.

## References

[ece38079-bib-0001] Acharya, P. R. , Bumrungsri, S. , & Racey, P. A. (2011). Cave Nectar Bat (*Eonycteris Spelaea*: Pteropodidae) Crucial Pollinator of Tropical Crops: Issues of Habitat Management and Conservation Problems. Proceeding of Asian Trans‐Disciplinary Karst Conference, 284–289.

[ece38079-bib-0002] Andrianaivoarivelo, R. A. , Andriafidison, D. , Rahaingonirina, C. , Raharimbola, S. , Rakotoarivelo, A. , Ramilijaona, O. R. , Racey, P. A. , & Jenkins, R. K. (2011). A conservation assessment of *Rousettus madagascariensis* (G. Grandidier, 1928, Pteropodidae) roosts in eastern Madagascar. Madagascar Conservation and Development, 6, 78–82.

[ece38079-bib-0003] Anthony, B. P. , Tatayah, V. , & De Chazal, D. (2018). Taking the first steps: Initial mapping of the human‐wildlife interaction of the Mauritius Fruit Bat *Pteropus niger* (Mammalia: Chiroptera: Pteropodidae) in Mauritius by conservation organizations. Journal of Threatened Taxa, 10, 12073–12081.

[ece38079-bib-0004] Aston, H. I. (1987). Influx of the grey‐headed flying‐fox *Pteropus poliocephalus* (Chiroptera: *Pteropodidae*) to the Melbourne area, Victoria, in 1986. Victorian Naturalist, 104, 9–13.

[ece38079-bib-0005] Aziz, S. A. , Olival, K. J. , Bumrungsri, S. , Richards, G. C. , & Racey, P. A. (2016). The conflict between pteropodid bats and fruit growers: Species, legislation and mitigation. In C. C. Voigt , & T. Kingston (Eds.), Bats in the Anthropocene: Conservation of bats in a changing world (pp. 377–426). Springer.

[ece38079-bib-0006] Bartholomew, G. A. , Leitner, P. , & Nelson, J. E. (1964). Body temperature, oxygen consumption, and heart rate in three species of Australian flying foxes. Physiological Zoology, 37, 179–198. 10.1086/physzool.37.2.30152330

[ece38079-bib-0007] BBC News Australia (2017). Australian town driven batty by flying foxes. BBC News Australia.

[ece38079-bib-0008] Birt, P. , & Markus, N. (1999). Notes on the temporary displacement of *Pteropus alecto* and *P. poliocephalus* by *P. scapulatus* within a daytime campsite. Australian Mammalogy, 21, 107–110.

[ece38079-bib-0009] Carroll, J. B. , & Feistner, A. T. (1996). Conservation of western Indian Ocean fruit bats. Biogéographie De Madagascar, 1996, 329–335.

[ece38079-bib-0010] Commonwealth of Australia (2015). Referral guideline for management actions in grey‐headed and spectacled flying‐fox camps (pp. 1–16). Commonwealth of Australia.

[ece38079-bib-0011] Commonwealth of Australia (2017a). Draft National Recovery Plan for the Grey‐headed Flying‐fox *Pteropus poliocephalus* . Commonwealth of Australia.

[ece38079-bib-0012] Commonwealth of Australia (2017b). Living with fruit bats: Inquiry into flying‐fox management in the eastern states. Commonwealth of Australia.

[ece38079-bib-0013] Connell, K. (2003). Population composition and diurnal behavioural patterns of the grey‐headed flying fox, *Pteropus poliocephalus* (Chiroptera: Pteropodidae), at roost sites in Sydney, NSW during autumn and winter. Honours Thesis. University of Technology.

[ece38079-bib-0014] Council of Ipswich (2016). Ipswich flying‐fox roost management plan (pp. 1–78). Council of Ipswich.

[ece38079-bib-0015] Crawley, M. J. (2013). The R book (2nd edn). John Wiley & Sons.

[ece38079-bib-0016] Currey, K. , Kendal, D. , van der Ree, R. , & Lentini, P. E. (2018). Land manager perspectives on conflict mitigation strategies for urban flying‐fox camps. Diversity, 10, 39. 10.3390/d10020039

[ece38079-bib-0017] Department of Agriculture Water and the Environment, A. G . (1999). Environment Protection and Biodiversity Conservation Act 1999. Department of Agriculture Water and the Environment.

[ece38079-bib-0018] Department of Environment and Primary Industries State Government of Victoria (1988). Flora and Fauna Guarantee Act 1988. Department of Environment and Primary Industries State Government of Victoria.

[ece38079-bib-0019] Department of Environment and Science (2020a). Code of Practice: Ecologically sustainable management of flying‐fox roosts. Department of Environment and Science.

[ece38079-bib-0020] Department of Environment and Science (2020b). Code of Practice: Low impact activities affecting flying‐fox roosts. Department of Environment and Science.

[ece38079-bib-0021] Eby, P. (1991). Seasonal movements of grey‐headed flying‐foxes, *Pteropus poliocephalus* (Chiroptera: Pteropodidae), from two maternity camps in northern New South Wales. Wildlife Research, 18, 547–559. 10.1071/WR9910547

[ece38079-bib-0022] Eby, P. , & Lunney, D. (2002a). Managing the Grey‐headed Flying‐fox Pteropus poliocephalus as a threatened species in NSW: Adjusting to a long‐term vision. Royal Zoological Society of New South Wales.

[ece38079-bib-0023] Eby, P. , & Lunney, D. (2002b). Managing the grey‐headed flying‐fox Pteropus poliocephalus as a threatened species: a context for the debate. In P. Eby , & D. Lunney (Eds.), Managing the Grey‐headed Flying‐fox as a Threatened Species in New South Wales (pp. 1–15). Royal Zoological Society of New South Wales.

[ece38079-bib-0024] Eby, P. , & Palmer, C. (1991) Flying‐foxes in rainforest remnants. In S. S. Phillips (Ed.), Proceedings of a Rainforest Remnant Rehabilitation Workshop held at Wollongbar, N.S.W. National Parks and Wildlife Service.

[ece38079-bib-0025] Eby, P. , Richards, G. , Collins, L. , & Parry‐Jones, K. (1999). The distribution, abundance and vulnerability to population reduction of a nomadic nectarivore, the grey‐headed flying‐fox *Pteropus poliocephalus* in New South Wales, during a period of resource concentration. Australian Zoologist, 31, 240–253.

[ece38079-bib-0026] EcoLogical (2014). Kareela flying‐fox camp plan of management. Prepared for Sutherland Shire Council (pp. 1–100). EcoLogical.

[ece38079-bib-0027] Forsyth, D. M. , Scroggie, M. P. , & McDonald‐Madden, E. (2006). Accuracy and precision of grey‐headed flying‐fox (*Pteropus poliocephalus*) flyout counts. Wildlife Research, 33, 57–65. 10.1071/WR05029

[ece38079-bib-0028] Fujita, M. S. , & Tuttle, M. D. (1991). Flying foxes (Chiroptera: Pteropodidae): Threatened animals of key ecological and economic importance. Conservation Biology, 5, 455–463. 10.1111/j.1523-1739.1991.tb00352.x

[ece38079-bib-0029] Geolink (2010). Final Maclean flying‐fox Management Strategy. Prepared for Clarence Valley Council and Department of Environment, Climate Change and Water on behalf of the Maclean flying‐fox Working Group.

[ece38079-bib-0030] Giles, J. R. , Eby, P. , Parry, H. , Peel, A. J. , Plowright, R. K. , Westcott, D. A. , & McCallum, H. (2018). Environmental drivers of spatiotemporal foraging intensity in fruit bats and implications for Hendra virus ecology. Scientific Reports, 8, 1–18. 10.1038/s41598-018-27859-3 29934514 PMC6015053

[ece38079-bib-0031] Giles, J. R. , Plowright, R. K. , Eby, P. , Peel, A. J. , & McCallum, H. (2016). Models of Eucalypt phenology predict bat population flux. Ecology and Evolution, 6, 7230–7245. 10.1002/ece3.2382 27891217 PMC5115174

[ece38079-bib-0032] Hahn, M. B. , Epstein, J. H. , Gurley, E. S. , Islam, M. S. , Luby, S. P. , Daszak, P. , & Patz, J. A. (2014). Roosting behaviour and habitat selection of *Pteropus giganteus* reveal potential links to Nipah virus epidemiology. Journal of Applied Ecology, 51, 376–387. 10.1111/1365-2664.12212 PMC4000083 24778457

[ece38079-bib-0033] Hahn, M. B. , Gurley, E. S. , Epstein, J. H. , Islam, M. S. , Patz, J. A. , Daszak, P. , & Luby, S. P. (2014). The role of landscape composition and configuration on *Pteropus giganteus* roosting ecology and Nipah virus spillover risk in Bangladesh. The American Journal of Tropical Medicine and Hygiene, 90, 247–255. 10.4269/ajtmh.13-0256 24323516 PMC3919225

[ece38079-bib-0034] Hall, L. S. (2002). Management of flying fox camps: What have we learnt in the last twenty five years. In P. Eby , & D. Lunney (Eds.), Managing the Grey‐headed Flying‐fox as a Threatened Species in NSW (pp. 215–224). Royal Zoological Society of New South Wales.

[ece38079-bib-0035] Hall, L. S. , & Richards, G. (2000). Flying foxes: Fruit and blossom bats of Australia. University of New South Wales Press.

[ece38079-bib-0036] Hodgkison, R. , Balding, S. T. , Zubaid, A. , & Kunz, T. H. (2003). Fruit Bats (Chiroptera: Pteropodidae) as seed dispersers and pollinators in a lowland Malaysian rain forest. Biotropica, 35, 491–502.

[ece38079-bib-0037] IUCN (2020). Red List. IUCN.

[ece38079-bib-0038] Jenkins, R. K. , Racey, P. A. , Andriafidison, D. , Razafindrakoto, N. , Razafimahatratra, E. , Rabearivelo, A. , Ratsimandresy, Z. , Andrianandrasana, R. H. , & Razafimanahaka, H. J. (2007). Not rare, but threatened: The endemic Madagascar flying fox *Pteropus rufus* in a fragmented landscape. Oryx, 41, 263–271.

[ece38079-bib-0039] Klose, S. M. , Welbergen, J. A. , Goldizen, A. W. , & Kalko, E. K. (2009). Spatio‐temporal vigilance architecture of an Australian flying‐fox colony. Behavioral Ecology and Sociobiology, 63, 371–380. 10.1007/s00265-008-0671-8

[ece38079-bib-0040] Kohut, T. (2017). Bat invasion in Australia town prompts closures, protests: ‘Kids are locked indoors’. Global News.

[ece38079-bib-0041] Kung, N. Y. , Field, H. E. , McLaughlin, A. , Edson, D. , & Taylor, M. (2015). Flying‐foxes in the Australian urban environment—community attitudes and opinions. One Health, 1, 24–30. 10.1016/j.onehlt.2015.07.002 28616461 PMC5441369

[ece38079-bib-0042] Ku‐ring‐gai Council (2018). Ku‐ring‐gai flying‐fox reserve 10 year site management and roosting habitat plan (pp. 1‐132). Ku‐ring‐gai Council.

[ece38079-bib-0043] Larsen, E. , Beck, M. , Hartnell, E. , & Creenaune, M. (2002). Neighbours of Ku‐ring‐gai Flying‐fox Reserve: Community Attitudes Survey 2001. In P. Eby , & D. Lunney (Eds.), Managing the Grey‐headed Flying‐fox as a Threatened Species in NSW (pp. 225–239). Royal Zoological Society of NSW.

[ece38079-bib-0044] Laurinec, P. (2017). Doing magic and analyzing seasonal time series with GAM (Generalized Additive Model) in R. Time series data mining in R.

[ece38079-bib-0045] Logan City Council (2015). Logan City Council flying‐fox management strategy 2015–2018 (pp. 1–20). Logan City Council.

[ece38079-bib-0046] Loughland, R. A. (1998). Mangal roost selection by the flying‐fox *Pteropus alecto* (Megachiroptera: Pteropodidae). Marine and Freshwater Research, 49, 351–352. 10.1071/MF97142

[ece38079-bib-0047] Lunney, D. , & Moon, C. (1997). Flying‐foxes and their camps in the rainforest remnants of north‐east NSW. Australia’s everchanging forests III (pp. 247–277). Centre for Resource and Environmental Studies, Australian National University.

[ece38079-bib-0048] Markus, N. (2002). Behaviour of the black flying fox *Pteropus alecto*: 2: Territoriality and Courtship. Acta Chiropterologica, 4, 153–166.

[ece38079-bib-0049] Markus, N. , & Blackshaw, J. K. (2002). Behaviour of the black flying fox *Pteropus alecto*: 1. An ethogram of behaviour, and preliminary characterisation of mother‐infant interactions. Acta Chiropterologica, 4, 137–152.

[ece38079-bib-0111] McIlwee, A. P. , & Martin, L. (2002). On the intrinsic capacity for increase of Australian flying‐foxes (*Pteropus* spp., Megachiroptera). Australian Zoologist, 32(1), 76–100.

[ece38079-bib-0050] McWilliam, A. N. (1984). The Gordon fruit bat colony Sydney: A report for the National Parks and Wildlife Service of the NSW Government. New South Wales State Government.

[ece38079-bib-0051] Meade, J. , Martin, J. M. , & Welbergen, J. A. (2021). Fast food in the city? Nomadic flying‐foxes commute less and hang around for longer in urban areas. Behavioral Ecology, 1–12.33708004

[ece38079-bib-0052] Meade, J. , van der Ree, R. , Stepanian, P. M. , Westcott, D. A. , & Welbergen, J. A. (2019). Using weather radar to monitor the number, timing and directions of flying‐foxes emerging from their roosts. Scientific Reports, 9, 1–10. 10.1038/s41598-019-46549-2 31308411 PMC6629676

[ece38079-bib-0053] Mickleburgh, S. P. , Hutson, A. M. , & Racey, P. A. (2002). A review of the global conservation status of bats. Oryx, 36, 18–34. 10.1017/S0030605302000054

[ece38079-bib-0054] Mo, M. , Roache, M. , Lenson, D. , Thomson, H. , Jarvis, M. , Foster, N. , Radford, A. , Oliver, L. , Oliver, D. L. , & Bentley, J. (2020). Congregations of a threatened species: Mitigating impacts from grey‐headed flying‐fox *Pteropus poliocephalus* camps on the Batemans Bay community. Australian Zoologist, 41, 124–138.

[ece38079-bib-0055] Mo, M. , Roache, M. , Williams, R. , Drinnan, I. N. , & Noël, B. (2020). From cleared buffers to camp dispersal: Mitigating impacts of the Kareela flying‐fox camp on adjacent residents and schools. Australian Zoologist, 41. 10.7882/AZ.2020.002

[ece38079-bib-0056] National Flying‐Fox Monitoring Program (2017). National flying‐fox monitoring viewer. Monitoring flying‐fox populations. National Flying‐Fox Monitoring Program.

[ece38079-bib-0057] Natural Earth (2020). Downloads: small scale data 1:110m. Natural Earth.

[ece38079-bib-0058] Nelson, J. (1965a). Movements of Australian flying foxes (Pteropodidae: Megachiroptera). Australian Journal of Zoology, 13, 53–74. 10.1071/ZO9650053

[ece38079-bib-0059] Nelson, J. E. (1965b). Behaviour of Australian Pteropodidae (Megacheroptera). Animal Behaviour, 13, 544–557. 10.1016/0003-3472(65)90118-1 5882814

[ece38079-bib-0060] New South Wales Government (2016). Biodiversity Conservation Act 2016. New South Wales Government.

[ece38079-bib-0061] Oleksy, R. , Racey, P. A. , & Jones, G. (2015). High‐resolution GPS tracking reveals habitat selection and the potential for long‐distance seed dispersal by Madagascan flying foxes *Pteropus rufus* . Global Ecology and Conservation, 3, 678–692. 10.1016/j.gecco.2015.02.012

[ece38079-bib-0062] Pallin, B. N. (2000). Ku‐ring‐gai Flying‐fox Reserve: Habitat restoration project, 15 years on. Ecological Management and Restoration, 1, 10–20. 10.1046/j.1442-8903.2000.00003.x

[ece38079-bib-0063] Palmer, C. , & Woinarski, J. (1999). Seasonal roosts and foraging movements of the black flying fox (*Pteropus alecto*) in the Northern Territory: Resource tracking in a landscape mosaic. Wildlife Research, 26, 823–838. 10.1071/WR97106

[ece38079-bib-0064] Parry‐Jones, K. (1985). Winter flying‐fox colonies in southern NSW. Australian Zoologist, 22, 5–6. 10.7882/AZ.1985.004

[ece38079-bib-0065] Parry‐Jones, K. , & Augee, M. (1991). Food selection by grey‐headed flying foxes (*Pteropus poliocephalus*) occupying a summer colony site near Gosford, New South Wales. Wildlife Research, 18, 111–124. 10.1071/WR9910111

[ece38079-bib-0066] Parry‐Jones, K. , & Augee, M. (1992). Movements of grey‐headed flying foxes (*Pteropus poliocephalus*) to and from colony site on the central coast of New South Wales. Wildlife Research, 19, 331–339. 10.1071/WR9920331

[ece38079-bib-0067] Parry‐Jones, K. , & Augee, M. (2001). Factors affecting the occupation of a colony site in Sydney, New South Wales by the Grey‐headed Flying‐fox *Pteropus poliocephalus* (Pteropodidae). Austral Ecology, 26, 47–55.

[ece38079-bib-0068] Parsons, J. G. , Robson, S. K. , & Shilton, L. A. (2011). Roost fidelity in spectacled flying‐foxes Pteropus conspicillatus: implications for conservation and management. In The biology and conservation of Australasian bats (pp. 66–71). Royal Zoological Society of NSW.

[ece38079-bib-0069] Parsons, J. G. , Van der Wal, J. , Robson, S. K. A. , & Shilton, L. A. (2010). The implications of sympatry in the spectacled and grey headed flying‐fox, *Pteropus conspicillatus* and *P. poliocephalus* (Chiroptera: Pteropodidae). Acta Chiropterologica, 12, 301–309.

[ece38079-bib-0070] Peel, A. J. , Wood, J. L. N. , Baker, K. S. , Breed, A. C. , Carvalho, A. D. , Fernández‐Loras, A. , Gabrieli, H. S. , Gembu, G.‐C. , Kakengi, V. A. , Kaliba, P. M. , Kityo, R. M. , Lembo, T. , Mba, F. E. , Ramos, D. , Rodriguez‐Prieto, I. , Suu‐Ire, R. , Cunningham, A. A. , & Hayman, D. T. S. (2017). How does Africa's most hunted bat vary across the continent? Population traits of the straw‐coloured fruit bat (*Eidolon helvum*) and its interactions with humans. Acta Chiropterologica, 19, 77–92. 10.3161/15081109ACC2017.19.1.006

[ece38079-bib-0071] Puddicombe, R. (1981). A Behavioural Study of the Greyheaded Flying‐fox, *Pteropus poliocephalus* (Megachiroptera). Honours Thesis. University of New England.

[ece38079-bib-0072] Queensland Government (1992). Nature Conservation Act 1992. Parliamentary Counsel State of Queensland.

[ece38079-bib-0073] Ratcliffe, F. N. (1931). The flying fox (*Pteropus*) in Australia. Commonwealth of Australia, Council for Scientific and Industrial Research Bulletin, 53, 1–81.

[ece38079-bib-0074] Ratcliffe, F. (1932). Notes on the fruit bats (*Pteropus* spp.) of Australia. The Journal of Animal Ecology, 1, 32–57. 10.2307/993

[ece38079-bib-0075] Richards, G. (2002). The development of strategies for management of the flying‐fox colony at the Royal Botanic Gardens, Sydney. In P. Eby , & D. Lunney (Eds.), Managing the Grey‐headed Flying‐fox as a Threatened Species in NSW (pp. 196–201). Royal Zoological Society of New South Wales.

[ece38079-bib-0076] Roberts, B. J. (2005). Habitat characteristics of flying‐fox roosts in south‐east Queensland. B.Sc. (Hons) Thesis. Griffith University.

[ece38079-bib-0077] Roberts, B. J. , Catterall, C. P. , Eby, P. , & Kanowski, J. (2012a). Latitudinal range shifts in Australian flying‐foxes: A re‐evaluation. Austral Ecology, 37, 12–22. 10.1111/j.1442-9993.2011.02243.x

[ece38079-bib-0078] Roberts, B. J. , Catterall, C. P. , Eby, P. , & Kanowski, J. (2012b). Long‐distance and frequent movements of the flying‐fox *Pteropus poliocephalus*: Implications for management. PLoS One, 7, e42532. 10.1371/journal.pone.0042532 22880021 PMC3411823

[ece38079-bib-0079] Scenic Rim Regional Council (2015). Flying‐fox management strategy (pp. 1–42). Scenic Rim Regional Council.

[ece38079-bib-0080] Schneider, A. , Friedl, M. A. , & Potere, D. (2009). A new map of global urban extent from MODIS satellite data. Environmental Research Letters, 4, 44003. 10.1088/1748-9326/4/4/044003

[ece38079-bib-0081] SEQ Catchments (2012). Management and restoration of flying‐fox camps: Guidelines and recommendations (pp. 1–32). SEQ Catchments. https://www.environment.nsw.gov.au/‐/media/OEH/Corporate‐Site/Documents/Animals‐and‐plants/Wildlife‐management/Flying‐foxes/management‐restoration‐flying‐fox‐camps.pdf?la=en&hash=0C1EB421162E0519A012B4BCE2BC438B20F6F01C

[ece38079-bib-0082] Shilton, L. A. , Altringham, J. D. , Compton, S. G. , & Whittaker, R. J. (1999). Old World fruit bats can be long–distance seed dispersers through extended retention of viable seeds in the gut. Proceedings of the Royal Society of London. Series B: Biological Sciences, 266, 219–223.

[ece38079-bib-0083] Shilton, L. A. , Latch, P. J. , McKeown, A. , Pert, P. , & Westcott, D. A. (2008). Landscape‐scale redistribution of a highly mobile threatened species, *Pteropus conspicillatus* (Chiroptera, Pteropodidae), in response to Tropical Cyclone Larry. Austral Ecology, 33, 549–561. 10.1111/j.1442-9993.2008.01910.x

[ece38079-bib-0084] Sinclair, E. , Webb, N. , Marchant, A. , & Tidemann, C. (1996). Genetic variation in the little red flying‐fox *Pteropus scapulatus* (Chiroptera: Pteropodidae): Implications for management. Biological Conservation, 76, 45–50. 10.1016/0006-3207(95)00086-0

[ece38079-bib-0085] Snoyman, S. , & Brown, C. (2010). Microclimate preferences of the grey‐headed flying fox (*Pteropus poliocephalus*) in the Sydney region. Australian Journal of Zoology, 58, 376–383. 10.1071/ZO10062

[ece38079-bib-0086] Stager, K. E. , & Hall, L. S. (1983). A cave‐roosting colony of the black flying‐fox (*Pteropus alecto*) in Queensland, Australia. Journal of Mammalogy, 64, 523–525. 10.2307/1380372

[ece38079-bib-0087] State of NSW and Department of Planning Industry and Environment (2019). Flying‐fox Camp Management Plan Template 2019. State of NSW and Department of Planning Industry and Environment.

[ece38079-bib-0088] State of NSW and Office of Environment and Heritage (2018). Flying‐fox camp management policy 2015 (pp. 1–22). Office of Environment and Heritage.

[ece38079-bib-0089] State of Queensland Department of Environment and Science (2020). Flying‐fox roost management guideline (pp. 1–50). State of Queensland Department of Environment and Science.

[ece38079-bib-0090] Sunshine Coast Regional Council (2016). Regional flying‐fox management plan: Environmental operations (pp. 1–72). Sunshine Coast Regional Council.

[ece38079-bib-0091] Tait, J. , Perotto‐Baldivieso, H. L. , McKeown, A. , & Westcott, D. A. (2014). Are flying‐foxes coming to town? Urbanisation of the spectacled flying‐fox (*Pteropus conspicillatus*) in Australia. PLoS One, 9, e109810. 10.1371/journal.pone.0109810 25295724 PMC4190360

[ece38079-bib-0092] Thiriet, D. (2010). Flying fox conservation laws, policies and practices in Australia: A case study in conserving unpopular species. Australasian Journal of Natural Resources Law and Policy, 13, 161.

[ece38079-bib-0093] Tidemann, C. R. (1999). Biology and management of the grey‐headed flying‐fox, *Pteropus poliocephalus* . Acta Chiropterologica, 1, 151–164.

[ece38079-bib-0094] Tidemann, C. R. , & Nelson, J. E. (2004). Long‐distance movements of the grey‐headed flying fox (*Pteropus poliocephalus*). Journal of Zoology, 263, 141–146. 10.1017/S0952836904004960

[ece38079-bib-0095] Tidemann, C. R. , Vardon, M. J. , Loughland, R. A. , & Brocklehurst, P. J. (1999). Dry season camps of flying‐foxes (Pteropus spp.) in Kakadu World Heritage Area, north Australia. Journal of Zoology, 247, 155–163. 10.1111/j.1469-7998.1999.tb00979.x

[ece38079-bib-0096] Trewhella, W. , Rodriguez‐Clark, K. , Corp, N. , Entwistle, A. , Garrett, S. , Granek, E. , Lengel, K. , Raboude, M. , Reason, P. , & Sewall, B. (2005). Environmental education as a component of multidisciplinary conservation programs: Lessons from conservation initiatives for critically endangered fruit bats in the western Indian Ocean. Conservation Biology, 19, 75–85. 10.1111/j.1523-1739.2005.00548.x

[ece38079-bib-0097] Van der Ree, R. , McDonnell, M. , Temby, I. , Nelson, J. , & Whittingham, E. (2006). The establishment and dynamics of a recently established urban camp of flying foxes (*Pteropus poliocephalus*) outside their geographic range. Journal of Zoology, 268, 177–185. 10.1111/j.1469-7998.2005.00005.x

[ece38079-bib-0098] Vardon, M. J. , Brocklehurst, P. S. , Woinarski, J. C. , Cunningham, R. B. , Donnelly, C. F. , & Tidemann, C. R. (2001). Seasonal habitat use by flying‐foxes, *Pteropus alecto* and *P. scapulatus* (Megachiroptera), in monsoonal Australia. Journal of Zoology, 253, 523–535. 10.1017/S0952836901000486

[ece38079-bib-0099] Vardon, M. J. , & Tidemann, C. R. (1999). Flying‐foxes (Pteropus alecto and P. scapulatus) in the Darwin region, north Australia: Patterns in camp size and structure. Australian Journal of Zoology, 47, 411–423. 10.1071/ZO99022

[ece38079-bib-0100] Welbergen, J. A. (2005). The social organisation of the grey‐headed flying‐fox, *Pteropus poliocephalus* . Doctor of Philosophy. University of Cambridge.

[ece38079-bib-0101] Welbergen, J. A. (2008). Variation in twilight predicts the duration of the evening emergence of fruit bats from a mixed‐species roost. Animal Behaviour, 75, 1543–1550. 10.1016/j.anbehav.2007.10.007

[ece38079-bib-0102] Welbergen, J. A. , Klose, S. M. , Markus, N. , & Eby, P. (2008). Climate change and the effects of temperature extremes on Australian flying‐foxes. Proceedings of the Royal Society of London B: Biological Sciences, 275, 419–425. 10.1098/rspb.2007.1385 PMC2596826 18048286

[ece38079-bib-0103] Welle, D. (2021). EcoWatch. https://www.ecowatch.com/flying‐foxes‐australia‐2649780401.html?rebelltitem=1#rebelltitem1

[ece38079-bib-0104] Westcott, D. A. , Caley, P. , Heersink, D. K. , & McKeown, A. (2018). A state‐space modelling approach to wildlife monitoring with application to flying‐fox abundance. Scientific Reports, 8, 1–9. 10.1038/s41598-018-22294-w 29511249 PMC5840426

[ece38079-bib-0105] Westcott, D. A. , & McKeown, A. (2004). Observer error in exit counts of flying‐foxes (*Pteropus* spp.). Wildlife Research, 31, 551–558. 10.1071/WR03091

[ece38079-bib-0106] Westcott, D. A. , McKeown, A. , Murphy, H. T. , & Fletcher, C. S. (2011). A monitoring method for the grey‐headed flying‐fox, *Pteropus poliocephalus* . CSIRO.

[ece38079-bib-0107] Williams, N. S. , Mcdonnell, M. J. , Phelan, G. K. , Keim, L. D. , & Van Der Ree, R. (2006). Range expansion due to urbanization: Increased food resources attract Grey‐headed Flying‐foxes (*Pteropus poliocephalus*) to Melbourne. Austral Ecology, 31, 190–198. 10.1111/j.1442-9993.2006.01590.x

[ece38079-bib-0108] Wood, S. N. (2017). Generalized additive models: An introduction with R. CRC Press.

[ece38079-bib-0109] Wood, S. W. , Prior, L. D. , Stephens, H. C. , & Bowman, D. M. J. S. (2015). Macroecology of Australian tall eucalypt forests: Baseline data from a continental‐scale permanent plot network. PLoS One, 10, e0137811. 10.1371/journal.pone.0137811 26368919 PMC4569531

[ece38079-bib-0110] Yang, L. , Qin, G. , Zhao, N. , Wang, C. , & Song, G. (2012). Using a generalized additive model with autoregressive terms to study the effects of daily temperature on mortality. BMC Medical Research Methodology, 12, 165. 10.1186/1471-2288-12-165 23110601 PMC3549928

