# Supplementary material for: Conventional wisdom on roosting behavior of Australian flying‐foxes—A critical review, and evaluation using new data
Source: Ecol Evol. 2021 Sep 9;11(19):13532–58. doi: 10.1002/ece3.8079 (PMC8495814; doi:10.1002/ece3.8079)
Supplement: Supplementary file 1 — Appendix S1‐S5 [file ECE3-11-13532-s001.docx]

**Supporting Information**

**Appendix S1-S5**

**Conventional wisdom on roosting behaviour of Australian flying foxes – a critical review, and evaluation using new data**

Tamika Lunn, Peggy Eby, Remy Brooks, Hamish McCallum, Raina Plowright, Maureen Kessler, Alison Peel

Author for correspondence:

Tamika J. Lunn

Email: tamika.lunn@griffithuni.edu.au

Appendix S1: Supporting Materials and Methods

**Details of literature search**

Table S1: Key words and combinations for the systematic literature search of peer-reviewed published literature using ISI’s Web of Knowledge

| *1* | *“Pteropus” OR “flying-fox” OR “flying fox” OR “fruit-bat” OR “fruit bat”* |
| --- | --- |
| *2* | *“Australia” OR “Queensland” OR “New South Wales” OR “Northern Territory” OR “Victoria” OR “South Australia” OR “Western Australia” OR “Australian Capital Territory” OR “Tasmania”* |
| *3* | *“Roost*” OR “Camp*”* |
| *COMBINED: (1 AND 2 AND 3)* | |

**Bibliography of literature included in review**

Birt, P. & Markus, N. (1999) Notes on the temporary displacement of *Pteropus alecto* and *P. poliocephalus* by *P. scapulatus* within a daytime campsite. *Australian Mammalogy,* **21,** 107-110.

Connell, K. (2003) Population composition and diurnal behavioural patterns of the grey-headed flying fox, *Pteropus poliocephalus* (Chiroptera: Pteropodidae), at roost sites in Sydney, NSW during autumn and winter. Honours Thesis, University of Technology.

Eby, P. (1991) Seasonal movements of grey-headed flying-foxes, *Pteropus poliocephalus* (Chiroptera: Pteropodidae), from two maternity camps in northern New South Wales. *Wildlife Research,* **18,** 547-559.

Eby, P. & Lunney, D. (2002) *Managing the Grey-headed flying-fox Pteropus poliocephalus as a threatened species in NSW: adjusting to a long-term vision*. Royal Zoological Society of New South Wales, Mosman, Australia.

Eby, P. & Palmer, C. (1991) Flying-foxes in rainforest remnants. *Proceedings of a Rainforest Remnant Rehabilitation Workshop held at Wollongbar, N.S.W.* (ed. S.S. Phillips). National Parks and Wildlife Service.

Eby, P., Richards, G., Collins, L. & Parry-Jones, K. (1999) The distribution, abundance and vulnerability to population reduction of a nomadic nectarivore, the grey-headed flying-fox *Pteropus poliocephalus* in New South Wales, during a period of resource concentration. *Australian Zoologist,* **31,** 240-253.

Forsyth, D.M., Scroggie, M.P. & McDonald-Maddena, E. (2006) Accuracy and precision of grey-headed flying-fox (*Pteropus poliocephalus*) flyout counts. *Wildlife Research,* **33,** 57-65.

Giles, J.R., Plowright, R.K., Eby, P., Peel, A.J. & McCallum, H. (2016) Models of Eucalypt phenology predict bat population flux. *Ecology and Evolution,* **6,** 7230-7245.

Hall, L.S. (2002) *Management of flying fox camps: what have we learnt in the last twenty five years*. Royal Zoological Society of New South Wales, Mosman, NSW.

Hall, L.S. & Richards, G. (2000) *Flying foxes: fruit and blossom bats of Australia*. University of New South Wales Press, Sydney, Australia.

Klose, S., Welbergen, J., Goldizen, A. & Kalko, E. (2009) Spatio-temporal vigilance architecture of an Australian flying-fox colony. *Behavioral Ecology and Sociobiology,* **63,** 371-380.

Larsen, E., Beck, M., Hartnell, E. & Creenaune, M. (2002) *Neighbours of Ku-ring-gai Flying-fox Reserve: Community Attitudes Survey 2001*. Royal Zoological Society of NSW, Mosman, Australia.

Loughland, R.A. (1998) Mangal roost selection by the flying-fox *Pteropus alecto* (Megachiroptera : Pteropodidae). *Marine and Freshwater Research,* **49,** 351-352.

Lunney, D. & Moon, C. (1997) Flying foxes and their camps in the remnant rainforests of north-east New South Wales. *Australia’s Everchanging Forests III: Proceedings of the Third National Conference on Australian Forest History* (ed. J. Dargavel), pp. 247-277. Centre for Resource and Environmental Studies, Australian National University, Canberra, Australia.

Markus, N. (2002) Behaviour of the black flying fox *Pteropus alecto*: 2. Territoriality and courtship. *Acta Chiropterologica,* **4,** 153-166.

Markus, N. & Blackshaw, J.K. (2002) Behaviour of the black flying fox *Pteropus alecto*: 1. An ethogram of behaviour, and preliminary characterisation of mother-infant interactions. *Acta Chiropterologica,* **4,** 137-152.

McWilliam A.N. (1984) The Gordon fruit bat colony Sydney: A report for the National Parks and Wildlife Service of the NSW Government. New South Wales State Government, Sydney, Australia.

Meade, J., van der Ree, R., Stepanian, P.M., Westcott, D.A. & Welbergen, J.A. (2019) Using weather radar to monitor the number, timing and directions of flying-foxes emerging from their roosts. *Scientific Reports,* **9**.

Nelson, J. (1965a) Movements of Australian flying foxes (Pteropodidae: Megachiroptera). *Australian Journal of Zoology,* **13,** 53-74.

Nelson, J.E. (1965b) Behaviour of Australian Pteropodidae (Megacheroptera). *Animal Behaviour,* **13,** 544-557.

Pallin, B.N. (2000) Ku‐ring‐gai Flying‐fox Reserve: Habitat restoration project, 15 years on. *Ecological Management & Restoration,* **1,** 10-20.

Palmer, C. & Woinarski, J.C.Z. (1999) Seasonal roosts and foraging movements of the black flying fox (*Pteropus alecto*) in the Northern Territory: resource tracking in a landscape mosaic. *Wildlife Research,* **26,** 823-838.

Parry-Jones, K. (1985) Winter flying-fox colonies in southern NSW. *Australian Zoologist,* **22,** 5-6.

Parry-Jones, K. & Augee, M. (1992) Movements of grey-headed flying foxes (*Pteropus poliocephalus*) to and from colony site on the central coast of New South Wales. *Wildlife Research,* **19,** 331-339.

Parry‐Jones, K. & Augee, M. (2001) Factors affecting the occupation of a colony site in Sydney, New South Wales by the Grey‐headed Flying‐fox *Pteropus poliocephalus* (Pteropodidae). *Austral Ecology,* **26,** 47-55.

Parsons, J.G., Robson, S.K.A. & Shilton, L.A. (2011) *Roost fidelity in spectacled flying-foxes Pteropus conspicillatus: implications for conservation and management*.

Parsons, J.G., Van der Wal, J., Robson, S.K.A. & Shilton, L.A. (2010) The implications of sympatry in the spectacled and grey headed flying-fox, *Pteropus conspicillatus* and *P. poliocephalus* (Chiroptera: Pteropodidae). *Acta Chiropterologica,* **12,** 301-309.

Puddicombe, R. (1981) A Behavioural Study of the Greyheaded Flying-fox, *Pteropus poliocephalus* (Megochiroptera). Honours Thesis, University of New England.

Ratcliffe, F. (1932) Notes on the fruit bats (*Pteropus* spp.) of Australia. *The Journal of Animal Ecology,* **1,** 32-57.

Ratcliffe, F.N. (1931) The flying fox (*Pteropus*) in Australia. *Commonwealth of Australia, Council for Scientific and Industrial Research Bulletin,* **53,** 1-81.

Richards, G. (2002) The development of strategies for management of the flying-fox colony at the Royal Botanic Gardens, Sydney. *Managing the Grey-headed Flying-fox as a Threatened Species in NSW* (eds P. Eby & D. Lunney), pp. 196-201. Royal Zoological Society of New South Wales, Mosman NSW.

Roberts, B.J. (2005) Habitat characteristics of flying-fox roosts in south-east Queensland. B.Sc. (Hons) thesis, Griffith University.

Roberts, B.J., Catterall, C.P., Eby, P. & Kanowski, J. (2012a) Latitudinal range shifts in Australian flying-foxes: A re-evaluation. *Austral Ecology,* **37,** 12-22.

Roberts, B.J., Catterall, C.P., Eby, P. & Kanowski, J. (2012b) Long-distance and frequent movements of the flying-fox *Pteropus poliocephalus*: implications for management. *Plos One,* **7**.

Shilton, L.A., Latch, P.J., McKeown, A., Pert, P. & Westcott, D.A. (2008) Landscape-scale redistribution of a highly mobile threatened species, *Pteropus conspicillatus* (Chiroptera, Pteropodidae), in response to Tropical Cyclone Larry. *Austral Ecology,* **33,** 549-561.

Snoyman, S. & Brown, C. (2010) Microclimate preferences of the grey-headed flying fox (*Pteropus poliocephalus*) in the Sydney region. *Australian Journal of Zoology,* **58,** 376-383.

Stager, K.E. & Hall, L.S. (1983) A cave-roosting colony of the black flying-fox (*Pteropus alecto*) in Queensland, Australia. *Journal of Mammalogy,* **64,** 523-525.

Tait, J., Perotto-Baldivieso, H.L., McKeown, A. & Westcott, D.A. (2014) Are flying-foxes coming to town? Urbanisation of the spectacled flying-fox (*Pteropus conspicillatus*) in Australia. *Plos One,* **9**.

Tidemann, C.R. (1999) Biology and management of the grey-headed flying-fox, *Pteropus poliocephalus*. *Acta Chiropterologica,* **1,** 151-164.

Tidemann, C.R. & Nelson, J.E. (2004) Long-distance movements of the grey-headed flying fox (*Pteropus poliocephalus*). *Journal of Zoology,* **263,** 141-146.

Tidemann, C.R., Vardon, M.J., Loughland, R.A. & Brocklehurst, P.J. (1999) Dry season camps of flying-foxes (*Pteropus spp*.) in Kakadu World Heritage Area, north Australia. *Journal of Zoology,* **247,** 155-163.

van der Ree, R., McDonnell, M.J., Temby, I., Nelson, J. & Whittingham, E. (2006) The establishment and dynamics of a recently established urban camp of flying foxes (*Pteropus poliocephalus*) outside their geographic range. *Journal of Zoology,* **268,** 177-185.

Vardon, M.J., Brocklehurst, P.S., Woinarski, J.C.Z., Cunningham, R.B., Donnelly, C.F. & Tidemann, C.R. (2001) Seasonal habitat use by flying-foxes, *Pteropus alecto* and *P-scapulatus* (Megachiroptera), in monsoonal Australia. *Journal of Zoology,* **253,** 523-535.

Vardon, M.J. & Tidemann, C.R. (1999) Flying-foxes (*Pteropus alecto* and *P-scapulatus*) in the Darwin region, north Australia: patterns in camp size and structure. *Australian Journal of Zoology,* **47,** 411-423.

Welbergen, J.A. (2005) The social organisation of the grey-headed flying-fox, *Pteropus poliocephalus*. University of Cambridge.

Welbergen, J.A. (2008) Variation in twilight predicts the duration of the evening emergence of fruit bats from a mixed-species roost. *Animal Behaviour,* **75,** 1543-1550.

Westcott, D.A., Caley, P., Heersink, D.K. & McKeown, A. (2018) A state-space modelling approach to wildlife monitoring with application to flying-fox abundance. *Scientific Reports,* **8**.

Westcott, D.A. & McKeown, A. (2004) Observer error in exit counts of flying-foxes (*Pteropus spp.*). *Wildlife Research,* **31,** 551-558.

Williams, N.S.G., McDonnell, M.J., Phelan, G.K., Keim, L.D. & Van der Ree, R. (2006) Range expansion due to urbanization: Increased food resources attract Grey-headed Flying-foxes (*Pteropus poliocephalus*) to Melbourne. *Austral Ecology,* **31,** 190-198.

**Detailed methods of empirical data collection**

*Empirical data collection*

Roost tree mapping

At each site, the spatial arrangement of all overstory, canopy and midstory trees were mapped in a grid network of 10 stratified random subplots (20 x 20 meters each). Subplots were stratified throughout perceived “core” (five subplots) and “peripheral” (five subplots) roosting areas, classed as areas observed to be frequently occupied (core) or infrequently (peripheral) by bats (Welbergen 2005). Core and peripheral areas were evaluated from regular observations made prior to roost tree mapping, though note that these categories were revised subsequently with the quantitative data. Trees were mapped using tree survey methods described in the “Ausplots Forest Monitoring Network, Large Tree Survey Protocol” (Wood et al. 2015). Briefly, this involved creating a grid network of subplots that were georeferenced at one corner. Distances were measured from the N/S or E/W subplot boundaries using an ultrasound distance instrument (Vertex Hypsometer, Haglöf Sweden, accurate to 10-30 cm) along the defined orientation bearing (Figure S1). Trees within the subplot were then mapped with the X-Y coordinate in relation to the georeferenced corner (0,0). To achieve maximum accuracy with the Vertex Hypsometer, only distances of up to 10 meters were recorded. If a tree was greater than 10 meters from the west/south origin (0 meter) subplot boundary, the tree was measured from the opposite (20 meter) subplot boundary, and the measured distance subtracted from 20 to give the distance from the origin boundary (Figure S1). Each tree was individually tagged and assigned a crown class following definitions in the Ausplots survey protocol (Wood et al. 2015) (Figure S2).

(Y=2m)

(X=5m)

(X=8m)

(X=20-8m)

Figure S1: Subplot layout for roost tree mapping. Subplots were oriented so that the left and right boundaries ran south to north, and the upper and bottom boundaries ran west to east. The origin (0,0) was situated on the southwest corner. Tree locations were mapped as X-Y coordinates (in meters) in relation to the origin (0,0) (examples in red: dots = trees, dotted lines = X and Y distances from the subplot boundaries). In this way, the western boundary gave the origin for the X coordinate, and the southern boundary for the Y coordinate. If a tree was greater than 10 meters from the west/south origin subplot boundary (0 meter), the tree was measured from the opposite east/north subplot boundary (20 meter), and the measured distance subtracted from 20 to give the distance from the origin boundary.


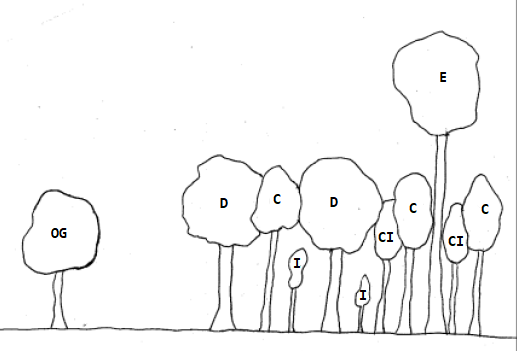


Overstory

Canopy

Midstory

Figure S2: Crown class categories for trees. Figure modified from the “Ausplots Forest Monitoring Network, Large Tree Survey Protocol.” For analysis, Emergent trees (E) were classified as “overstory”, Dominant (D), Co-dominant (C) and Open Ground (OG) trees were classified as “canopy”, and Intermediate (I) and sub-Canopy Intermediate (CI) were classified as “midstory”.

Roosting surveys

To evaluate spatio-temporal patterns in roosting, we revisited all tagged trees and scored the extent of species occupancy using the following index: 0= zero bats; 1= 1-5 bats; 2=6-10 bats; 3=11-20 bats; 4=21-50 bats; 5=51-100 bats, 6=101-200 bats, 7= >200 bats. For a subset of trees (N=60 per site) absolute counts and minimum/maximum roosting heights of each species were taken. Roosting heights were measured using the Vertex Hypsometer. The tree subset was randomly selected, stratified by subplot and crown class. Stratifications included six trees per subplot, with one tree in each crown class except emergent, from which two trees were chosen. If a specific crown class was not present in a subplot, the next crown class down in height was selected instead. The selected random subset of trees was consistent through time.

All observations were made from a distance to minimise potential disturbance to bats during the survey, and to prevent bats moving away during counting efforts: tagged trees were located with the aid of roost maps showing the locations of subplots, and tree maps showing the relative X-Y coordinate of trees within subplots. Tree tags and bats were viewed through binoculars. Height measurements were conducted after counting, as this involved attaching an ultrasound unit to the trunk of the tree of interest, and so had a higher potential for disturbing the flying-foxes. Original positions of the lowest and highest roosting animals (relative to prominent branches) were noted during counting in case animals became disturbed. In general, bats showed minimal response to the observers during the surveys, providing they remained quiet, did not move quickly, and kept an appropriate distance. This is consistent with other studies on flying-foxes, which have noted that bats rarely move from their roosting position unless they are disturbed by noise or sudden activity, and that the diurnal activities of flying-foxes consist predominantly of sedentary activities like roosting, sleeping, social interactions and grooming (Markus & Blackshaw 2002). Most disturbances elicit minimal responses (e.g. hanging tensely, or swaying to view the source of disturbance), but larger disturbances can result in scrambling or taking flight to avoid perceived threats. In the latter case, individuals typically return to their roosting locations 10-20 minutes once the disturbance has ceased (Markus & Blackshaw 2002). At some of our sites, the animals were also habituated to regular human presence along pathways underneath the roost (habituation is easily accomplished in flying-foxes (Welbergen 2006; Klose *et al.* 2009a)).

Overall roost perimeter was mapped with GPS (accurate to 10 meters) immediately after the tree survey to estimate perimeter length and roost area. The roost perimeter boundary was defined as per (Clancy & Einoder 2004), by walking directly underneath roosting flying-foxes, to delineate between inhabited and uninhabited roosting habitat. The area around the roost was also searched to ensure that there were no substantial “satellite” roosts outside the defined area of interest. The GPS perimeter was then overlayed with a satellite map of the site, and the total area and location of the roost centre (centroid) was calculated in QGIS 3.1. Total abundance at each roost was estimated with a census count of bats where feasible (i.e. where total abundance was predicted to be <5,000 individuals), or by counting bats as they emerged in the evening from their roosts (“fly-out”), as per recommendations in Westcott *et al.* (2011). If these counts could not be conducted, population counts from local councils (conducted within roughly a week of the bat surveys) were used, as total abundance of roosts are generally stable over short timeframes (Nelson 1965b). Because roost estimates become more unreliable with increasing abundance, we also converted the total estimated abundance into an index estimate, as per values used by the National Flying-fox Monitoring Program (National Flying-Fox Monitoring Program 2017). Index categories were as follows: 1 = 1-499 bats; 2 = 500-2,499 bats; 3 = 2,500 - 4,999 bats; 4 = 5,000 - 9,999 bats; 5 = 10,000 - 15,999 bats; 6 = 16,000 - 49,999 bats; and 7 = > 50,000 bats. Roosting surveys were repeated once a month for 13 months (August 2018 - August 2019).

**Detailed methods of empirical data analysis**

*Statistical analyses*

Model structure

We utilised generalized additive models for all statistical comparisons to allow for nonlinearity in the time-series, with random effects (session, roost site and subplot) modelled with smooth functions. A summary of these comparisons and corresponding model structures is given in Table S2, and a breakdown of spatial and temporal replicates of measures is given in Table S3.

Table S2: Summary of model structure for each comparison. Note that error distribution for comparisons were specified according to data type and extent of zero-inflation. Choice of distribution therefore depended on whether only occupied subplots/trees were included in the comparison (not zero-inflated), or both occupied and unoccupied subplots/trees (zero-inflated).

| **Comparison** | **Response variable** | **Independent variable(s)** | **Distribution** | **Link function** | **Random effects** | **Seasonal time-series** | **Species** |
| --- | --- | --- | --- | --- | --- | --- | --- |
| Use of area | | | | | | | |
| Determine whether bat occupation is greater for subplots in ‘core’ areas of the roost compared with subplots in irregularly occupied ‘peripheral’ areas. | Total number of bats per occupied subplot | Plot type (peripheral or core) | Gamma | Log | Subplot nested with roost site per session | Yes | Combined into one measure |
|  | Proportion of occupied trees per occupied subplot | Plot type (peripheral or core) | Gaussian | Log | Subplot nested with roost site per session | Yes | Combined into one measure |
| Density of bats in occupied core and peripheral trees | Total number of bats per occupied tree | Tree type (peripheral or core) | Gamma | Log | Subplot nested with roost site per session | Yes | Combined into one measure |
| Determine whether bat occupation decreases with distance from the roost centre | Total number of bats per subplot (occupied or non- occupied) | Distance from the roost centre (centroid) | Poisson | Log | Subplot nested with roost site plus session | Yes | Separated |
|  | Proportion of occupied trees per subplot (occupied or non- occupied) | Distance from the roost centre (centroid) | Gaussian | Identity | Subplot nested with roost site plus session | Yes | Separated |
| Demographic/social structure | | | | | | | |
| Determine whether dominant individuals occupy the centre of roosts, and subdominant individuals the outer area | Proportion of male bats per tree (as a proxy indicator of dominance groupings) | Distance from the roost centre (centroid) | Gaussian | Identity | Subplot nested with roost site plus session | No | Separated |
| Sympatry of species | | | | | | | |
| Differences in roosting height between species, and relationship between roosting height and total abundance per tree | Maximum roosting height | Total number of bats per occupied tree | Gamma | Log | Subplot nested with roost site plus session | Yes | Separated |

Table S3: Spatial and temporal replicates of measures within the data. The maximum total number of datapoints for each measure can be calculated by the number of spatial replicates multiplied by the number of temporal replicates. Note that this is the maximum number of replicates – some measures could not be calculated where there were either no bats or a single bat in the tree (e.g. height range), and summary measures were calculated from occupied subplots and occupied trees where relevant (e.g. subplot-level total bat abundance). A full set of summary data, including a full breakdown of sample sizes, are given in Appendix S2.

|  | **Spatial replicates** | | | **Temporal replicates** |
| --- | --- | --- | --- | --- |
| **Measure** | **Overall** | **Per roost** | **Per subplot** |  |
| *Roost-level measure* | | | | |
| Total number of bats per roost (index) | 8 | 1 | NA | 13 |
| Total roost perimeter / area | 8 | 1 | NA | 13 |
| Proportion of subplots occupied per roost (all subplots) | 8 | 1 | NA | 13 |
| *Subplot-level measure* | | | | |
| Total number of bats per subplot (all subplots) | 80 | 10 | 1 | 13 |
| Proportion of occupied trees per subplot (all subplots) | 80 | 10 | 1 | 13 |
| *Tree-level measure* | | | | |
| Total number of bats per tree (index) | 2,522 | 118-474 | 2-75 | 13 |
| Total number of bats per tree (absolute count) | 480 | 60 | 6 | 13 |
| Minimum / maximum roosting height | 480 | 60 | 6 | 13 |
| Proportion of male bats per tree | 480 | 60 | 6 | 13 |

*Comparisons*

Use of area

To evaluate whether some areas of the roosts were more consistently occupied than others, we generated a histogram of subplot occupancy from all occupied roost sites Figure S3. From visual inspection of this histogram we determined a threshold of 80% occupancy to distinguish between ‘core’ and ‘peripheral’ subplots. The same approach was done to identify ‘core’ and ‘peripheral’ tree usage Figure S4, and the same threshold of 80% was reached. We thus refer to ‘core’ and ‘peripheral’ areas/trees, being subplots/trees occupied in at least 80% of surveys (‘core’) or less (‘peripheral’).

We then determined whether bat occupation is greater for subplots in ‘core’ areas of the roost compared with subplots in irregularly occupied ‘peripheral’ areas using the generalized additive modelling approach. This approach was repeated for different bat abundance metrics, including total number of bats per occupied subplot and proportion of occupied trees per occupied subplot. As difference between species was not of primary interest for this comparison, we combined species prior to analyses to give a combined bat abundance. We also used generalized additive modelling to determine whether bat occupation decreased with distance from the roost centre. For these models, we calculated distance as the average distance of trees per subplot (subplot-level metrics). Modelling followed the same approach as previously described, but with models split by species to allow differences in seasonality of occupation (and so, differences in the fit of cyclic cubic regression splines), and with the distance as the independent variable.


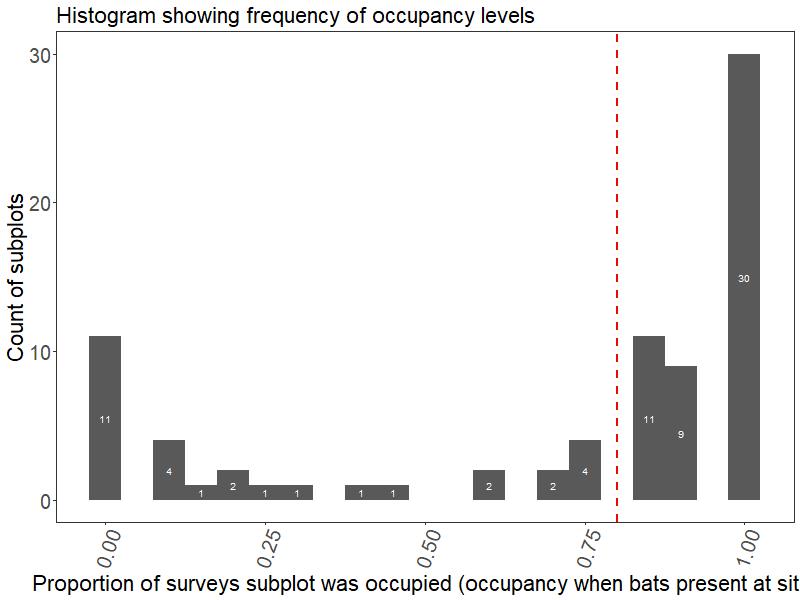


Figure S3: Histogram showing frequency of subplot occupancy. X-axis is the proportion of times each subplot was occupied, for surveys when at least one bat was present in the roost (bins=0.05), and y-axis and labels show the frequency of each bin.


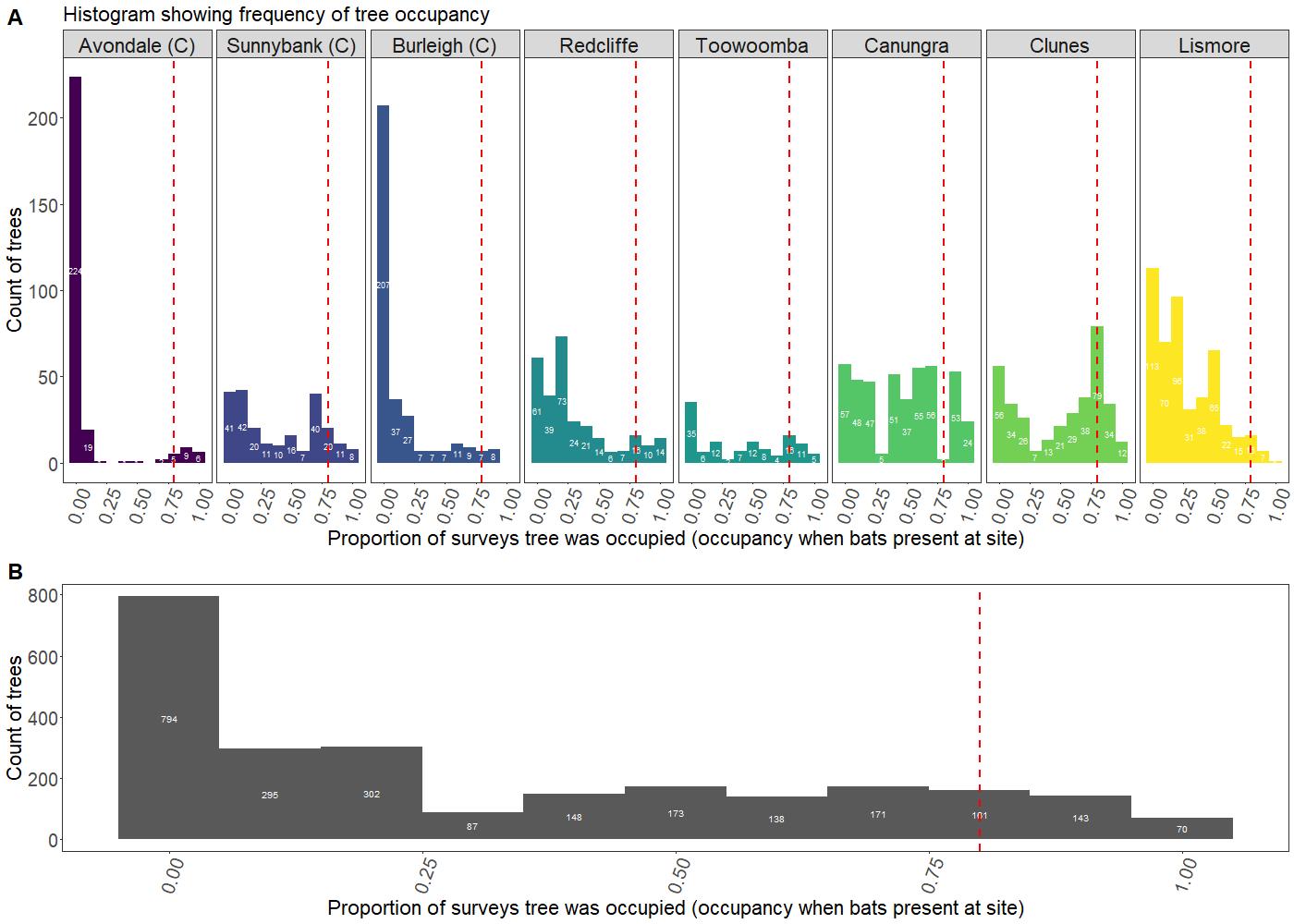


Figure S4: Frequency plot, of the proportion of times individual trees were occupied across surveys (for surveys when at least one bat was present in the roost). A) shows frequency of tree occupancy split by roost site (facet and colour), and B) shows frequency of tree occupancy across roosts combined. Numbers indicate the number of trees in each bin. Y-axis is the count of individual trees (for example, in A count of trees per facet will sum to the number of trees at the site, for B count of trees will sum to the total number of trees).

Sympatry of species

To evaluate the level of species sympatry within trees, we investigated differences in roosting height between species. Modelling followed the same approach as previously described, but with maximum roosting height as the response, and bat abundance per tree included as the independent effect. Models were split by species to allow differences in seasonality of occupation (and so, differences in the fit of cyclic cubic regression splines).

To evaluate within-roost sympatry of species, we report on the distribution and occupancy of species at each site over time. We provide interactive images of species kernel density and distribution through time in Appendix S4. Interactive images were produced in RMarkdown using the display function from the package EBImage (Pau *et al.* 2010).

Demographic/social structure

To investigate whether dominant individuals occupy the centre of roosts, and subdominant individuals the outer area, we use the proportion of male bats in trees as a proxy indicator of dominance groupings. We assume that dominant and non-dominant groups reflect mating and non-mating groups respectively, and hence that dominant groups are comprised of a low proportion of males (a single male among multiple females), and subdominant groups are comprised of a higher proportion of males (mixed non-breeding males) (Markus 2002). We modelled the proportion of male bats in occupied trees as a function of distance of individual trees from the roost centroid, with random effects of site, subplot and survey session, as previously described. We also provide a spatial maps of male groupings relative to the roost perimeter though time, in Appendix S5.

Roost abundance/occupancy

To evaluate seasonal patterns of abundance and occupancy, we show different scales of bat abundance measures through time, per roost site. Measures included the number of bats per occupied subplot, proportion of occupied trees in occupied subplots, total roost area, and total roost abundance, as well as the proportion of male bats per occupied tree through time.

Appendix S2: Summarised data

Online only interactive table, available at < https://doi.org/10.5061/dryad.g4f4qrfqv >

Appendix S3: Supporting Results

**Expanded table of statements and literature support**

Table S4: Common understandings in state-level documents. Note that additional evidence from our 13-month empirical study only addresses questions that require less than one year of data (i.e. intra-annual patterns in roost structure). Statements not addressed with our empirical data are coloured grey.

| **Understandings** | **Referenced by** | **Empirical evidence** | | **Additional evidence (this study)** | |
| --- | --- | --- | --- | --- | --- |
|  |  | **Support** | **Contradict** | **Support** | **Contradict** |
| ***Use of area:*** |  |  |  |  |  |
| Some areas of permanent camps are more consistently occupied ('core areas’) than others | SEQ Catchments (2012); EcoLogical (2014) | Welbergen (2005) – reasonably stable centre (defined as point of greatest density; varied by 4.8-23.8 meters). Ephemeral use of edge. Note that sampling was inter-annual but not intra-annual  Richards (2002) – refers to a ‘core’ group of residents, but does not indicate whether the location of core group was consistent through time  Nelson (1965b) – notes peripherally occupied edge areas (GHFF) |  | Figure 2 |  |
| ‘Core areas’ are more densely occupied than ‘peripheral areas’ | SEQ Catchments (2012) | Nelson (1965b) – increase in density from outside (1-5 animals per tree) to centre (up to 400 animals per tree) (GHFF)  Welbergen (2005) – high density at roost centre (8.7 bats/m^2^) and low density at edge (0-2 bats/m^2^) (GHFF & BFF) (though note this study selects the roost centre from the highest density value) |  | Figure 3; Figure 4; Appendix S3 |  |
| Roost area fluctuates with total abundance | SEQ Catchments (2012); EcoLogical (2014) | Welbergen (2005) – notes variation in roost area (1.06 and 1.35 ha) and abundance (26,500-30,800), but does not quantify association between the two (GHFF & BFF)  Pallin (2000) – notes variable abundance and area (2-3 ha), but does not formally quantify relationship (GHFF)  Larsen *et al.* (2002) – note that maximum roost extent is different to average summer extent, but do not formally quantify a relationship (GHFF) |  | Figure 5 |  |
| Flying-foxes adjust the location of ‘core areas’ through time | SEQ Catchments (2012) | Hall (2002) & Pallin (2000) – observed to occupy different areas over time (GHFF) | Welbergen (2005) – reasonably stable centre between years (but note, study only 2 years) |  |  |
| Areas outside of the 'core area' are used by more transient animals | SEQ Catchments (2012) | Welbergen (2005) – higher fidelity (“microsite tenure”) in the roost centre |  |  |  |
|  |  |  |  |  |  |
| **Spatial segregation of species:** |  |  |  |  |  |
| Species share roosts sites, but segregate spatially within | Commonwealth of Australia (2017a) | Welbergen (2005) – BFF roost in edge areas, GHFF in centre areas  Ratcliffe (1932) – LRFF and BFF noted to occupy different areas  Parsons *et al.* (2010) – note BFF, GHFF, SFF & LRFF roosting together in a single roost. Note that LRFF roosted separately within the roost  Nelson (1965b) – species usually form separate groups, but will hang together outside the reproductive season if numbers are low (GHFF, BFF, LRFF)  Klose *et al.* (2009b) – BFF roost in edge areas (when present), GHFF in centre areas | Parsons *et al.* (2010) – note BFF, GHFF, SFF & LRFF roosting together in a single roost. BFF, GHFF and SFF observed to share roost trees  Markus (2002) – BFF & GHH regularly seen to share particular roost trees in areas of overlap within a roost | Figure 6; Appendix S4 |  |
| Large influxes of species into roosts (especially little red flying-foxes) can displace other species |  | Birt and Markus (1999) – displacement of BFF & GHFF by LRFF |  | Appendix S4 |  |
| Species roost at different heights | Geolink (2010) | Welbergen (2005) – BFF roost in taller trees than GHFF. GHFF also roost lower in shared trees (measurement: number of bats per segment of tree)  Roberts (2005) – BFF roost higher (7 m - 12 m) than GHFF (5-9 m) and vertical stratification of species was commonly observed |  | Figure 7 |  |
| Indirect competition favours black flying-foxes over grey-headed flying-foxes | Commonwealth of Australia (2017a); EcoLogical (2014) | Ratcliffe (1931) – suggest BFF are relacing GHFF owing to increase in BFF but not GHFF | Markus (2002) – no evidence of aggression between BFF & GHFF  Roberts (2005) – competition not in the form of exclusion from roosting. BFF and GHFF observed to co-occupy roosts |  |  |
|  |  |  |  |  |  |
| **Demographic/social structure:** |  |  |  |  |  |
| The majority of roost trees are occupied by mixed groups of adults, with territories comprised of a single male and one or more females and their dependent young | SEQ Catchments (2012); State of Queensland Department of Environment and Science (2020) | Welbergen (2005) – seasonal mixed sex groups of a single male and up to 5 females (Jan-May) (GHFF)  Puddicombe (1981) – noted reproductive groups (mixed groups of males, females and their young)  Nelson (1965b) & Nelson (1965a) – mixed groups of reproducing animals in the breeding season (GHFF & BFF)  Markus and Blackshaw (2002) – note groups with mostly females with young and small harem groups, but also note branches with single male individuals (BFF)  Markus (2002) – seasonal mixed sex groups of a single male and up to 4 females (March-April) (GHFF & BFF). Lactating and non-lactating females occurred in these groups  Eby *et al.* (1999) – note mixed groups of adult males, adult females and subadults of both sexes  McWilliam (1984) – seasonal mixed sex groups of a single male and 2-5 females. Predominantly groups of mixed sex, with few territories of solitary males or sub-adult animals (GHFF)  Connell (2003) – note mixed sex groups but also territories of solitary males (GHFF) | Welbergen (2005) – note that sexes were segregated horizontally and vertically during nursing/before mating, but were together during and after mating (GHFF)  Nelson (1965b) & Nelson (1965a) – winter camps are sexually segregated and/or contain only juveniles/subadults. Sexes are segregated in summer camps between Sep-Dec (vertical or horizontal segregation) (GHFF & BFF) | Figure 8 |  |
| Dominant individuals (defined as reproducing males and females) occupy the centre of roosts and subdominant individuals (defined as non-reproducing males and females) the outer area | State of Queensland Department of Environment and Science (2020) | Nelson (1965b) – female groups larger in centres of colonies. Males roosting near the periphery of the colonies rarely roost with females. Assume that males without territories occur in peripheral areas (GHFF & BFF)  Welbergen (2005) – mating territories observed in the roost centre, solitary males observed at the edge. Females occupied roost centre during mating season only – i.e. horizontal segregation of sexes observed | Puddicombe (1981) – reproductive groups uniformly distributed through the roost  Markus and Blackshaw (2002) – note that areas are consistently occupied by different group types, but don’t describe spatial location |  | Figure 8; Appendix S5 |
| Individuals at the periphery of groups act as ‘guards’ | State of Queensland Department of Environment and Science (2020) | Nelson (1965b) – note ‘guard groups’ on the perimeter of the roost (GHFF)  Klose *et al.* (2009b) – demonstrate that vigilance is highest by bats at the roost periphery (GHFF) |  |  |  |
| Juveniles wean and leave their mothers from January and form groups on the edge of their existing roost or at another site | State of Queensland Department of Environment and Science (2020) |  | Welbergen (2005) – observation of specific ‘juvenile trees’ but distributed randomly across the roost. Also no effect of season on segregation of juveniles (GHFF)  Nelson (1965b) & Nelson (1965a) – note ‘juvenile packs’. Spatial location not described (GHFF & BFF)  Markus and Blackshaw (2002) – note a ‘sub-adult section’. Spatial location not described (BFF)  Eby *et al.* (1999) – note separate roosting of sub-adults but spatial location not described (GHFF)  Connell (2003) – note separate roosting of juveniles but spatial location not described (GHFF) |  |  |
| The roosting positions of individual males are highly consistent and animals return to the same branch of a tree over many weeks or months | SEQ Catchments (2012) | Welbergen (2005) – high “microsite faithfulness” (95.0 ±1.02 %) independent of sex but dependent on age*sex (adult males higher than adult females and young). Tenure of microsite was 27.6 ±3.25 days with a maximum recorded tenure of 137 days. Only adult males displayed between-year microsite faithfulness  Markus and Blackshaw (2002) – note that specific sites along individual branches were continuously occupied by animals of the same sex and body size and were assumed to be the same individuals  Markus (2002) – note territory establishment, plus an observation of consistent branch use by animals of the same sex and body size, and suggest that specific individuals show site fidelity over time within roosts | Tidemann and Nelson (2004) – variable roost occupancy by one male GHFF (consistent occupancy between 1-10 weeks)  Roberts *et al.* (2012b) – variable roost occupancy (14 male GHFF): 64% of roost visits lasted <5 consecutive days, but some individuals remained at one roost for several months  Parsons, Robson and Shilton (2011) – high turnover of SFF within trees |  |  |
|  |  |  |  |  |  |
| **Roost abundance/occupancy:** |  |  |  |  |  |
| Individual roosts have distinguishable seasonal patterns of abundance and occupation. | Abundance:  Commonwealth of Australia (2017a);  Occupation:  State of Queensland Department of Environment and Science (2020) | Abundance  Westcott *et al.* (2018) – lowest population April-Sept, highest Nov-Feb (SFF)  Welbergen (2005) – Population decrease from May, lowest in July-October (absent)  Tait *et al.* (2014) – lowest May-July, highest Jan-March (SFF)  Parry‐Jones and Augee (2001) – abundance highly cyclical with lowest population in July, highest in January (GHFF)  Parry-Jones and Augee (1992) – abundance matched cyclic occupancy, with peak abundance ~Jan-Feb (GHFF)  Nelson (1965b) & Nelson (1965a) – roost in small groups or individually in winter (GHFF)  Meade *et al.* (2019) – seasonal pattern within years, peak Feb-April and min July-August (GHFF)  Occupation  Roberts (2005) – Regularly used roosts occupied more frequently (80% occupancy) than ‘irregularly used’ roosts (30% occupancy) for roosts within south-east QLD (BFF & GHFF)  Welbergen (2005) – Main roost site not occupied July-October  Vardon and Tidemann (1999) – seasonal migration of BFF and LRFF to/from the Darwin region  Parry-Jones and Augee (1992) – seasonal occupation of GHFF. Absent July-September  Parry-Jones (1985) – overwintering of roost in rainforested valley (occupation June-Sep) (GHFF)  Nelson (1965b) & Nelson (1965a) – note two types of roosts: summer (Sep-April) and winter (April-Sep). Summer camps occupied consecutively across years, winter camps used inconsistently and over short duration (GHFF & BFF)  Nelson (1965b) & Nelson (1965a) – migration of LRFF southward Nov-April  Klose *et al.* (2009b) – Roost not occupied May-October (GHFF)  Puddicombe (1981) – LRFF southward in summer (December-February) | Abundance  Shilton *et al.* (2008) – SFF patterns strongly affected by extreme weather. Roosts smaller post cyclone  Richards (2002) – no relationship between abundance and time of year (GHFF)  Roberts (2005) – no difference in abundance between winter and summer (BFF & GHFF)  Occupation  Van der Ree *et al.* (2006) – continuous occupation of Melbourne roost from 1993 (GHFF)  Richards (2002) – continuous occupation of Sydney roost (GHFF)  Puddicombe (1981) – continuous occupation noted from 1981  Shilton *et al.* (2008) – SFF patterns strongly affected by extreme weather. Dispersed into new roosts post cyclone | Figure 9 |  |
| Intra- and inter-annual variations in abundance can be extreme | Commonwealth of Australia (2017a) | Westcott and McKeown (2004) – flyout counts varied by 20–247% (SFF)  Tait *et al.* (2014) – seasonal max ~20x higher than seasonal min (SFF)  Welbergen (2008) – variable fly-out counts (BFF & GHFF)  Welbergen (2005) – variable estimates, between 26,500 and 30,800 bats (BFF & GHFF)  Large (site specific) change in roost abundance within and between years: BFF & LRFF (Vardon & Tidemann 1999); LRFF (Ratcliffe 1931; Ratcliffe 1932); GHFF (Eby 1991; Eby & Palmer 1991; Eby & Lunney 2002a; Van der Ree *et al.* 2006)  Roberts *et al.* (2012a) – seasonal max ~50x higher than seasonal min, with relatively stable patterns between years (GHFF). Minimal seasonality to BFF abundance, but highly variable across years (50x higher in annual peak than min)  Richards (2002) – variable abundance, range 1000-6000 (GHFF)  Parry‐Jones and Augee (2001) – variable abundance, range ~10,000 – 50,000. Difference between seasonal min and max within years ~ 20,000 bats (GHFF)  Parry-Jones and Augee (1992) – variable abundance, range ~0 – 80,000 (GHFF)  Pallin (2000) – variable between year abundance, range from 100s to 80,000 (GHFF). Within year fluctuation between 13,000 (winter min) to 45,000 (summer max)  Meade *et al.* (2019) – large interannual variation (min record 10 ±7 bats, max record 46,169 ± 3,097), large intra-annual variation (difference between min and max within years ~20,000-40,000) (GHFF)  Loughland (1998) – substantial within-season variation (60-30,000 bats within a single dry season) (BFF)  Giles *et al.* (2016) (citing abundance data from the Queensland flying-fox monitoring program database) – large intra-annual variation (min ~100s – max 20,000), predicted by time-lagged changes of Eucalypt-focused vegetation indices (proxy for nectar availability)  Forsyth, Scroggie and McDonald-Madden (2006) – highly variable fly-out counts across a 2-day period (Day 1: 4,013 - Day 2: 949)  Eby *et al.* (1999) – variable fly-out counts across a 2-day period, but roost dependent (e.g. counts from one roost Day 1: 7,700 – Day 2: 400)  Lunney and Moon (1997) – describe anecdotal reports of historical roost abundance. Note variable patterns of occupation and abundance | Roberts (2005) – trends in abundance remained constant within and between years (BFF & GHFF) | Figure 9 |  |
| Roost abundance peaks in March | State of Queensland Department of Environment and Science (2020) | Van der Ree *et al.* (2006) (GHFF); Tait *et al.* (2014) (SFF); Meade *et al.* (2019) (GHFF); Eby (1991) (GHFF); Eby and Palmer (1991) (GHFF)  Nelson (1965a) – noted a peak in March-April for one roost (GHFF) | Westcott *et al.* (2018) – peak Nov-Feb (SFF)  Welbergen (2005) – peak in April (GHFF & BFF)  Vardon and Tidemann (1999) – timing of peaks variable between roosts and years, but ~May/July (BFF, Darwin region)  Vardon *et al.* (2001) – peak BFF: June-August (main) or January-March (satellite).  Roberts *et al.* (2012a) – two peaks per year: between Jan-Feb & August (GHFF). Fluctuation of BFF minimal  Richards (2002) – highest recorded abundance in October (GHFF)  Parry‐Jones and Augee (2001) – seasonal peaks in January (GHFF)  Parry-Jones and Augee (1992) – seasonal peaks ~Jan-Feb (GHFF)  Pallin (2000) (citing personal communication with M. Beck) – population peak in summer (GHFF)  Nelson (1965b) & Nelson (1965a) – peak in Dec-Jan (GHFF & BFF) |  | Figure 9; Appendix S3 |
| Consistent (inter-annual) patterns in abundance and use are more commonly observed in roosts located in 1) extensive areas of rainforest, and 2) urban areas | SEQ Catchments (2012); Commonwealth of Australia (2017a) | Extensive rainforest  Parry-Jones (1985) – overwintering of roost in rainforested valley (occupation June-Sep) (GHFF)  Urban areas  Tait *et al.* (2014) – urban-associated SFF roosts were more consistently occupied than non-urban roosts  Welbergen (2005) – Main roost site (with non-urbanised surroundings) not occupied July-October (BFF & GHFF)  Van der Ree *et al.* (2006) – continuous occupation of Melbourne roost from 1993 (GHFF)  Richards (2002) – continuous occupation of Sydney roost (GHFF)  Williams *et al.* (2006) – greater temporal availability of food in urban areas (Melbourne) (GHFF)  Parry‐Jones and Augee (2001) – continuous occupation of roost site in Sydney (GHFF)  Parry-Jones and Augee (1992) – seasonal occupation (non-overwintering) of roost in peri-urban area (GHFF) |  |  |  |
|  |  |  |  |  |  |
| **Habitat preferences:** |  |  | Stager and Hall (1983) – observation of cave roosting BFF |  |  |
| The habitat patch must be at least 1ha in size but be large enough to accommodate and sustain large numbers of flying-foxes. For a small roost (10,000 bats) the area needed is approximately 3ha and for a large roost (50,000) the area needed is 10ha | SEQ Catchments (2012); State of NSW and Department of Planning Industry and Environment (2019) as per State of NSW and Office of Environment and Heritage (2018); EcoLogical (2014) | Pallin (2000) – occupancy of 1-2 ha (GHFF)  Roberts (2005) – roosts within south-east QLD mostly located in vegetation at least 1ha in size |  |  |  |
| Flying-foxes prefer complex vegetation structure (upper, mid- and understorey layers) | SEQ Catchments (2012); State of NSW and Department of Planning Industry and Environment (2019) as per State of NSW and Office of Environment and Heritage (2018) | Pallin (2000) (citing report by Buchanan) – use vegetation consisting of four layers |  |  | |
| Flying-foxes prefer dense vegetation | SEQ Catchments (2012) | Roberts (2005) – Surveyed roosts ranged in basal area from 15 – 44 m^2^/ ha on average. Minimum basal area was 0-10 m^2^/ha and maximum > 83 m^2^/ha |  |  | |
| Flying-foxes prefer a dense understory | SEQ Catchments (2012) | Roberts (2005) – roosts within south-east QLD mostly have dense vegetation, unless inundated by water surfaces (e.g. mangroves). |  |  | |
| Flying-foxes prefer a closed canopy at least 3-5m high | SEQ Catchments (2012); State of NSW and Department of Planning Industry and Environment (2019) as per State of NSW and Office of Environment and Heritage (2018); EcoLogical (2014) | Tidemann *et al.* (1999) – minimum canopy height for BFF and LRFF roosts 6.7 m  Tidemann (1999) – GHFF can establish camps in most types of closed vegetation > 3 m in height  Roberts (2005) – choice of trees >10 m for roosting. No bats observed in trees <4 m | Welbergen (2005) – average height of trees occupied was between 11.6-17.6 meters. Showed no apparent preference for tree height |  | |
| The structure of roost-wide vegetation is more important than the characteristics of individual roost trees (e.g. species, canopy cover) | SEQ Catchments (2012) | Colonies occur in many different vegetation types (Palmer & Woinarski 1999; Tidemann *et al.* 1999; Vardon & Tidemann 1999; Pallin 2000; Vardon *et al.* 2001; Roberts 2005) (BFF, LRFF, GHFF)  Roberts (2005) – selecting roosting areas at three hierarchical levels: regional scale, then roost scale, then tree scale  Hall and Richards (2000) – vegetation structure potentially important for roost selection |  |  | |
| Flying-foxes prefer level topography (<5° incline) | SEQ Catchments (2012); State of NSW and Department of Planning Industry and Environment (2019) as per State of NSW and Office of Environment and Heritage (2018) | Roberts (2005) – preference for flat topography (BFF & GHFF) |  |  |  |
| Flying-foxes prefer to roost within 50 km of the coastline or at an elevation <65 m above sea level | SEQ Catchments (2012); State of NSW and Department of Planning Industry and Environment (2019) as per State of NSW and Office of Environment and Heritage (2018) | Hall and Richards (2000) – coastal distribution (BFF & GHFF)  Roberts (2005) – Of 40 studied roosts 26 were within 24 km of the coast. Three roosts found >65 km from the coast.  Roberts (2005) – 80% of study roosts located at altitudes less than 60 m and 62.5% less than 20 m | Ratcliffe (1931) – report roosts as far inland as Jandowae and Eidsvold (~200km inland)  Ratcliffe (1932) – usually not more than 80km inland otherwise |  |  |
|  |  |  |  |  |  |
| **Roost macroclimate:** |  |  |  |  |  |
| The mid-storey vegetation within roosts is critical for maintaining a cool, humid and sheltered environment that is stable against the outside environment | SEQ Catchments (2012); State of NSW and Department of Planning Industry and Environment (2019) as per State of NSW and Office of Environment and Heritage (2018) | Loughland (1998) – temperature at roosts lower than control areas (BFF) | Snoyman and Brown (2010) – humidity and temperature larger in roost than outside roost (attributed to defoliation within roost) |  | |
|  |  |  |  |  |  |
| **Negative impacts from flying-foxes:** |  |  |  |  |  |
| Impacts sustained over several years of flying-fox occupancy can lead to damage and/or death of individual roost trees | SEQ Catchments (2012); State of Queensland Department of Environment and Science (2020) | Welbergen (2005) – densely occupied centre areas stripped of leaves and small branches. Vegetation intact in edge areas (GHFF)  Richards (2002) – crown damage to roost trees  Pallin (2000) – note die-back of canopy trees before restoration interventions (GHFF)  McWilliam (1984) – note loss of leaf cover in areas with roosting bats (GHFF)  Hall (2002) – note extensive damage due to LRFF (collapse of whole trees under combined weight), but less damage from roosting GHFF (loss of leaf and bark cover) |  |  | |
| Some tree species are more resilient to damage by flying-fox roosting than others | SEQ Catchments (2012) |  |  |  | |
| In small remnant patches, the process of opening the canopy (from tree damage by roosting) will increase the impact of invasive weeds | SEQ Catchments (2012); State of Queensland Department of Environment and Science (2020) | Pallin (2000) – note weed infestation before restoration interventions (GHFF)  McWilliam (1984) & Hall (2002) – note weed infestation of roost, attributed to bats (GHFF) |  |  | |
| Where sufficient roosting space is available, flying-foxes shift their roosting areas, which lessens their damage to vegetation over time | SEQ Catchments (2012); EcoLogical (2014) | Pallin (2000) – observed to occupy different areas over time (GHFF)  Hall (2002) – note that shifting of roosting area by GHFF and LRFF allows damaged vegetation to regenerate and recover, usually within the next growing season |  |  | |

**Model output**

*Core/peripheral area comparison*

Response: number of bats per tree

Table S5: Model output of the general additive model. Model compares the number of bats per occupied subplot between core and peripheral subplots (occupied more than or less than 80% of surveys where bats were present, respectively)

| Nested General Additive Model with seasonal sessional term | | |
| --- | --- | --- |
| Variable | Coef (± se) | p |
| Fixed effects | | |
| Intercept | 5.281 (0.18) | 0 |
| Peripheral subplots | -0.581 (0.177) | 0.0011 |
| Random effects | | |
| Session |  | 0.7352 |
| Site |  | 0 |
| Subplot |  | 0.001 |

Response: Proportion of occupied trees

Table S6: Model output of the general additive model. Model compares the proportion of occupied trees per occupied subplot between core and peripheral subplots (occupied more than or less than 80% of surveys where bats were present, respectively)

| Nested General Additive Model with seasonal sessional term | | |
| --- | --- | --- |
| **Variable** | **Coef (± se)** | **p** |
| **Fixed effects** | | |
| Intercept | -0.836 (0.063) | 0 |
| Peripheral subplots | -0.222 (0.078) | 0.0047 |
| **Random effects** | | |
| Session |  | 0.0045 |
| Site |  | 0.0102 |
| Subplot |  | 0.0014 |

*Core/peripheral tree comparison*

Table S7: Model output of the general additive model. Model compares the number of bats per occupied tree between core and peripheral trees (occupied more than or less than 80% of surveys where bats were present, respectively)

| Nested General Additive Model with seasonal sessional term | | |
| --- | --- | --- |
| **Variable** | **Coef (± se)** | **p** |
| **Fixed effects** | | |
| Intercept | 2.966 (0.141) | 0 |
| Peripheral trees | -0.606 (0.034) | 0 |
| **Random effects** | | |
| Session |  | 0.1994 |
| Site |  | 0 |
| Subplot |  | 0 |

*Density/abundance with distance from roost centre*

Response: number of bats per plot

Table S8: Model output of the general additive models, first with species combined, then separated. Models evaluate the total number of bats per occupied subplot, as a function of distance from the roost centre

| Nested General Additive Model with seasonal sessional term | | |
| --- | --- | --- |
| **Variable** | **Coef (± se)** | **p** |
| **Fixed effects** | | |
| Intercept | 5.439 (0.224) | 0 |
| Distance from centroid | -1.672 (0.017) | 0 |
| **Random effects** | | |
| Session |  | 0 |
| Site |  | 0 |
| Subplot |  | 0 |

| Nested General Additive Model with seasonal sessional term - Black flying-fox | | |
| --- | --- | --- |
| **Variable** | **Coef (± se)** | **p** |
| **Fixed effects** | | |
| Intercept | 4.687 (0.191) | 0 |
| Distance from centroid | -1.286 (0.023) | 0 |
| **Random effects** | | |
| Session |  | 0 |
| Site |  | 0 |
| Subplot |  | 0 |

| Nested General Additive Model with seasonal sessional term - Grey-headed flying-fox | | |
| --- | --- | --- |
| **Variable** | **Coef (± se)** | **p** |
| **Fixed effects** | | |
| Intercept | 4.357 (0.415) | 0 |
| Distance from centroid | -1.893 (0.028) | 0 |
| **Random effects** | | |
| Session |  | 0 |
| Site |  | 0 |
| Subplot |  | 0 |

| Nested General Additive Model with seasonal sessional term - Little red flying-fox | | |
| --- | --- | --- |
| **Variable** | **Coef (± se)** | **p** |
| **Fixed effects** | | |
| Intercept | -1.658 (1.778) | 0.3528 |
| Distance from centroid | 0.725 (0.087) | 0 |
| **Random effects** | | |
| Session |  | 0 |
| Site |  | 0 |
| Subplot |  | 0 |

Response: Proportion of occupied trees

Table S9: Model output of the general additive model, first with species combined, then separated. Models evaluate the proportion of occupied trees per occupied subplot, as a function of distance from the roost centre

| Nested General Additive Model with seasonal sessional term | | |
| --- | --- | --- |
| **Variable** | **Coef (± se)** | **p** |
| **Fixed effects** | | |
| Intercept | 0.474 (0.0521) | 0 |
| Distance from centroid | -0.324 (0.034) | 0 |
| **Random effects** | | |
| Session |  | 6e-04 |
| Site |  | 0 |
| Subplot |  | 0.0065 |

| Nested General Additive Model with seasonal sessional term - Black flying-fox | | |
| --- | --- | --- |
| **Variable** | **Coef (± se)** | **p** |
| **Fixed effects** | | |
| Intercept | 0.283 (0.047) | 0 |
| Distance from centroid | -0.176 (0.028) | 0 |
| **Random effects** | | |
| Session |  | 0.002 |
| Site |  | 0 |
| Subplot |  | 0 |

| Nested General Additive Model with seasonal sessional term - Grey-headed flying-fox | | |
| --- | --- | --- |
| **Variable** | **Coef (± se)** | **p** |
| **Fixed effects** | | |
| Intercept | 0.318 (0.06) | 0 |
| Distance from centroid | -0.292 (0.034) | 0 |
| **Random effects** | | |
| Session |  | 0.721 |
| Site |  | 0 |
| Subplot |  | 0 |

| Nested General Additive Model with seasonal sessional term - Little red flying-fox | | |
| --- | --- | --- |
| **Variable** | **Coef (± se)** | **p** |
| **Fixed effects** | | |
| Intercept | 0.098 (0.039) | 0.0123 |
| Distance from centroid | -0.075 (0.061) | 0.2236 |
| **Random effects** | | |
| Session |  | 0.5721 |
| Site |  | 0.264 |
| Subplot |  | 3e-04 |

*Demographic/social structure*

Response: proportion of male bats per tree

Table S10: Model output of the general additive models, first with species combined, then separated. Models evaluates the proportion of male bats per tree, as a function of distance from the roost centre

| Nested General Additive Model with a seasonal sessional term | | |
| --- | --- | --- |
| **Variable** | **Coef (± se)** | **p** |
| **Fixed effects** | | |
| Intercept | 0.379 (0.023) | 0 |
| Distance from centroid | 0.15 (0.039) | 1e-04 |
| **Random effects** | | |
| Session |  | 0.5608 |
| Site |  | 0.0031 |
| Subplot |  | 0.0065 |

| Nested General Additive Model with a seasonal sessional term - Black flying-fox | | |
| --- | --- | --- |
| **Variable** | **Coef (± se)** | **p** |
| **Fixed effects** | | |
| Intercept | 0.413 (0.031) | 0 |
| Distance from centroid | 0.168 (0.05) | 7e-04 |
| **Random effects** | | |
| Session |  | 0.7051 |
| Site |  | 3e-04 |
| Subplot |  | 0.0113 |

| Nested General Additive Model with a seasonal sessional term - Grey-headed flying-fox | | |
| --- | --- | --- |
| **Variable** | **Coef (± se)** | **p** |
| **Fixed effects** | | |
| Intercept | 0.372 (0.03) | 0 |
| Distance from centroid | 0.02 (0.063) | 0.7524 |
| **Random effects** | | |
| Session |  | 0.6773 |
| Site |  | 0.2858 |
| Subplot |  | 0.1746 |

| Nested General Additive Model with seasonal sessional term - Little red flying-fox | | |
| --- | --- | --- |
| **Variable** | **Coef (± se)** | **p** |
| **Fixed effects** | | |
| Intercept | 0.422 (0.112) | 4e-04 |
| Distance from centroid | 0.011 (0.203) | 0.9588 |
| **Random effects** | | |
| Session |  | 0.091 |
| Site |  | 0.6195 |
| Subplot |  | 0.1957 |

*Roosting heights of species*

Response: maximum roosting height

Table S11: Model output of the general additive models. Models evaluate the maximum roosting heights of bats per tree, as a function of total bats per tree, first with species combined, then separated

| Nested General Additive Model with seasonal sessional term | | |
| --- | --- | --- |
| **Variable** | **Coef (± se)** | **p** |
| **Fixed effects** | | |
| Intercept | 2.697 (0.056) | 0 |
| Number of bats in tree | 0.004 (0) | 0 |
| **Random effects** | | |
| Session |  | 0 |
| Site |  | 0 |
| Subplot |  | 0.0073 |

| Nested General Additive Model with seasonal sessional term - Black flying-fox | | |
| --- | --- | --- |
| **Variable** | **Coef (± se)** | **p** |
| **Fixed effects** | | |
| Intercept | 2.791 (0.056) | 0 |
| Number of bats in tree | 0.004 (0) | 0 |
| **Random effects** | | |
| Session |  | 0.0103 |
| Site |  | 0 |
| Subplot |  | 0 |

| Nested General Additive Model with seasonal sessional term - Grey-headed flying-fox | | |
| --- | --- | --- |
| **Variable** | **Coef (± se)** | **p** |
| **Fixed effects** | | |
| Intercept | 2.542 (0.082) | 0 |
| Number of bats in tree | 0.006 (0) | 0 |
| **Random effects** | | |
| Session |  | 0.0018 |
| Site |  | 0 |
| Subplot |  | 3e-04 |

| Nested General Additive Model with seasonal sessional term - Little red flying-fox | | |
| --- | --- | --- |
| **Variable** | **Coef (± se)** | **p** |
| **Fixed effects** | | |
| Intercept | 2.476 (0.093) | 0 |
| Number of bats in tree | 0.001 (0) | 0.0054 |
| **Random effects** | | |
| Session |  | 0.1304 |
| Site |  | 0.0019 |
| Subplot |  | 0.2594 |

**Additional visuals of empirical data analysis**


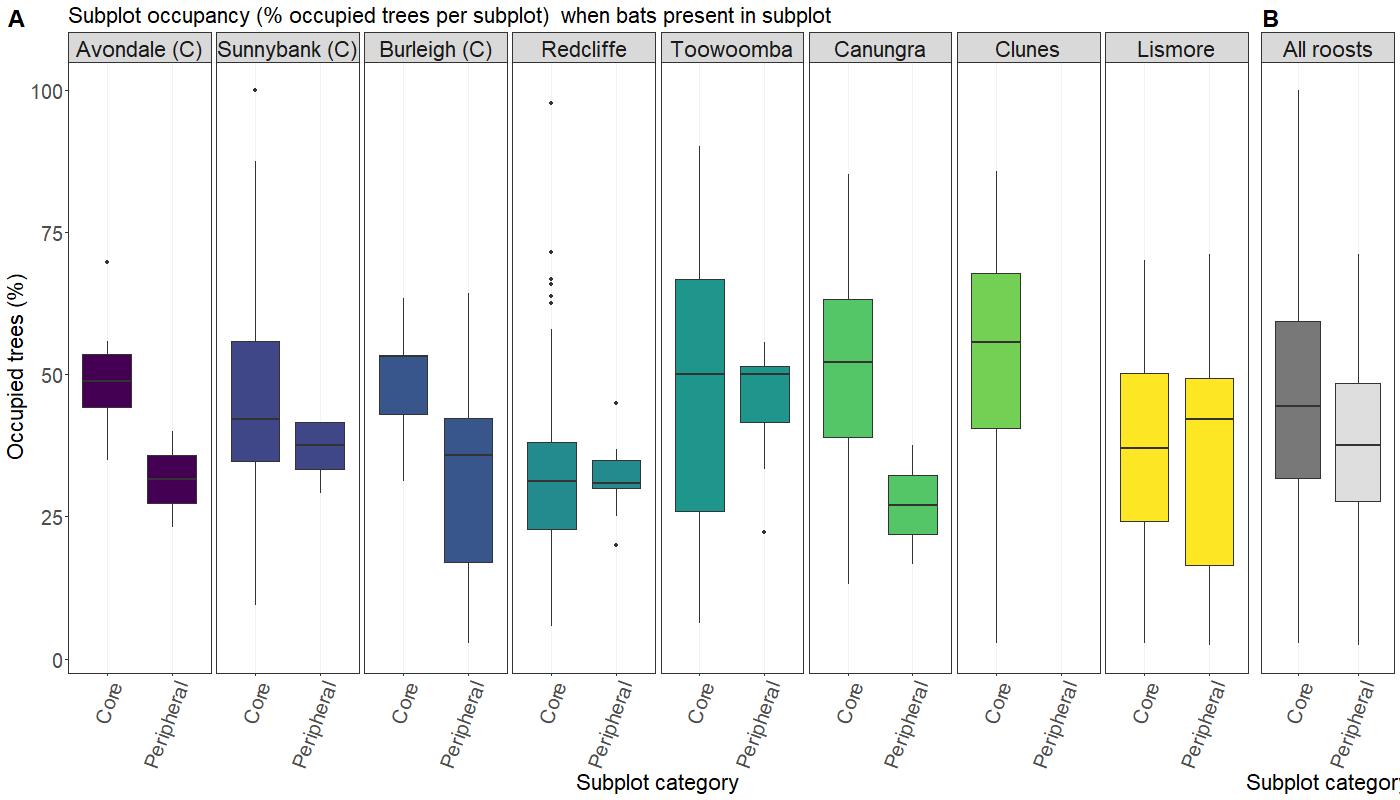


Figure S5: Occupancy of subplots in ‘core’ and ‘peripheral’ areas, shown by the total number of bats per subplot across the survey period. Number of bats is estimated from index values, where each tree is assigned a ‘weighted index value’ being the middle value of the index range. Data is filtered to show numbers of bats when subplots are occupied (i.e. unoccupied subplots are removed). ‘Core’ subplots were identified as those occupied in at least 80% of surveys (when bats present at the roost), and ‘peripheral’ subplots as those occupied less than 80% of the time. A) Shows areas split by roost site (facet and colour), and B) shows all roosts combined. Area displayed in plot has been cropped to remove outliers.


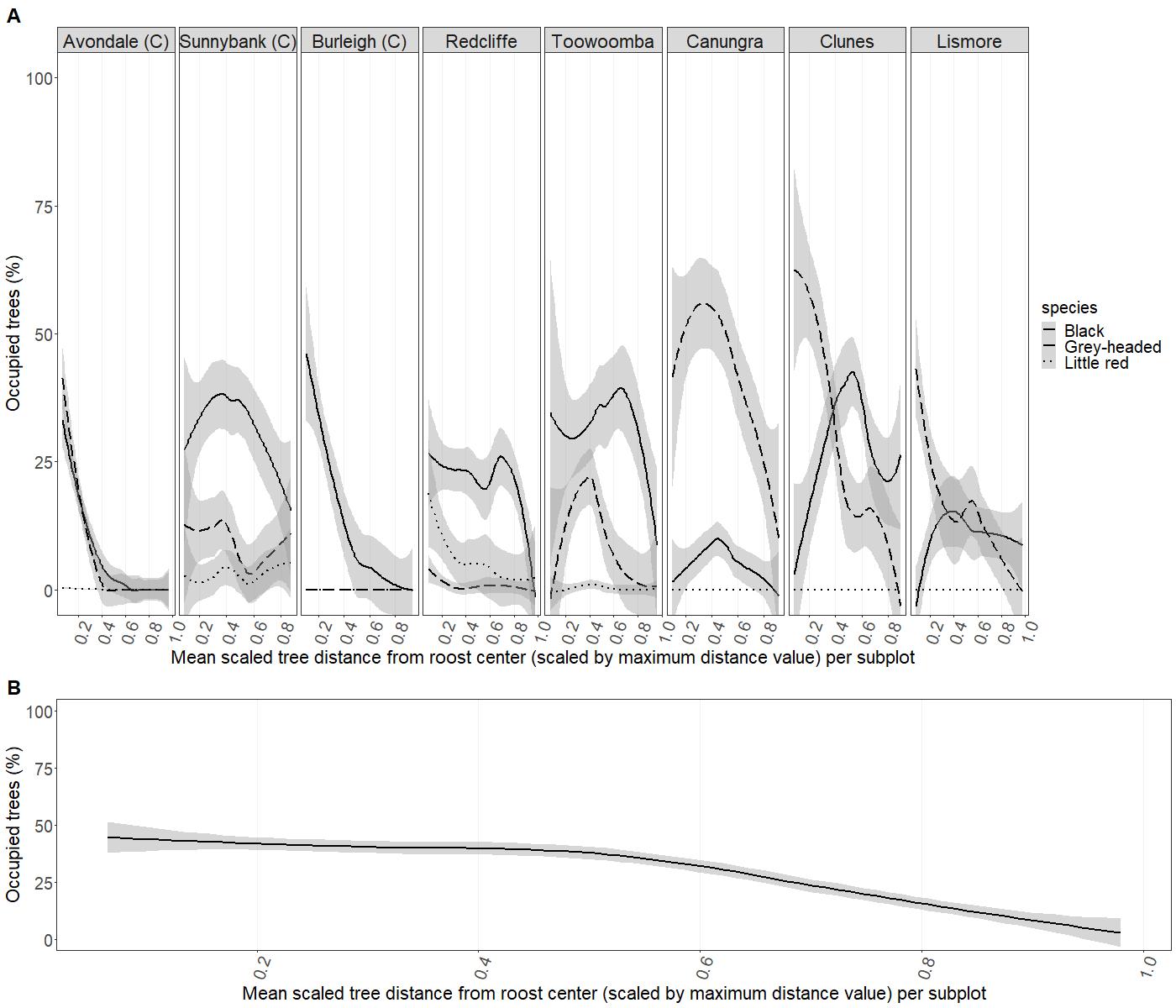


Figure S6: Distance from roost centre and occupancy of bats, shown by the proportion of occupied trees per subplot during the survey period. Data is filtered to show numbers of bats when trees are occupied (i.e. unoccupied subplots are removed). Roost centre is calculated for each survey, as the centroid of the roost area at the time of the survey. Distance from the centre is calculated as the mean distance of trees in each subplot from this centroid, scaled by the maximum observed distance value per session. A) shows values per species (line type) split by roost (facets); and B) shows species and roost combined. Area displayed in plot has been cropped to remove outliers. Trend line is by loess fit (local polynomial regression fit) with standard error bands (grey shading).


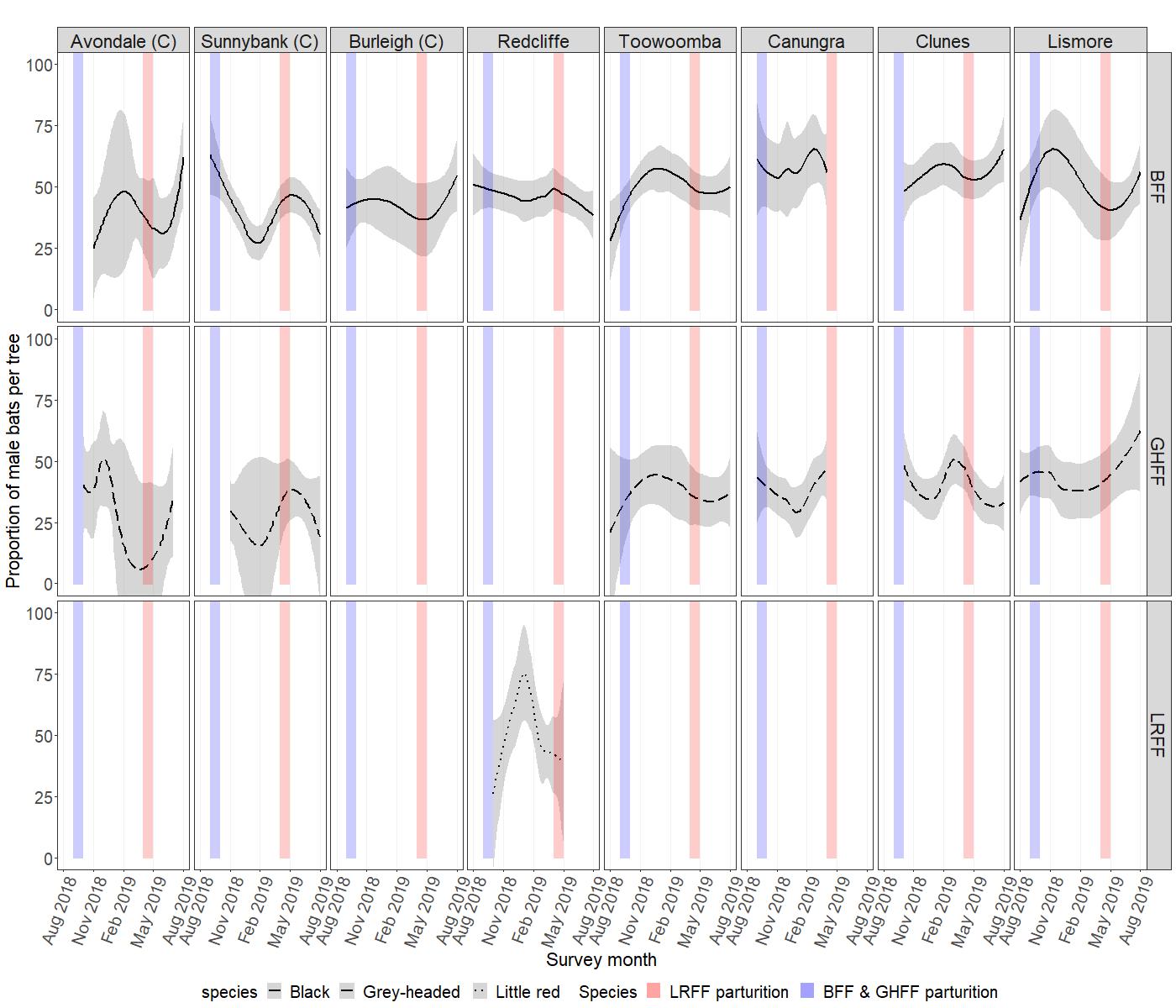


Figure S7: Proportion of male bats per occupied tree through time, facetted by roost site (columns) and species (rows). Trend line is by loess fit (local polynomial regression fit) with standard error bands (grey shading). Vertical blue shading shows approximate timing of parturition for grey-headed flying-foxes and black flying-foxes, and vertical red shading shows approximate timing of parturition for little red flying-foxes.


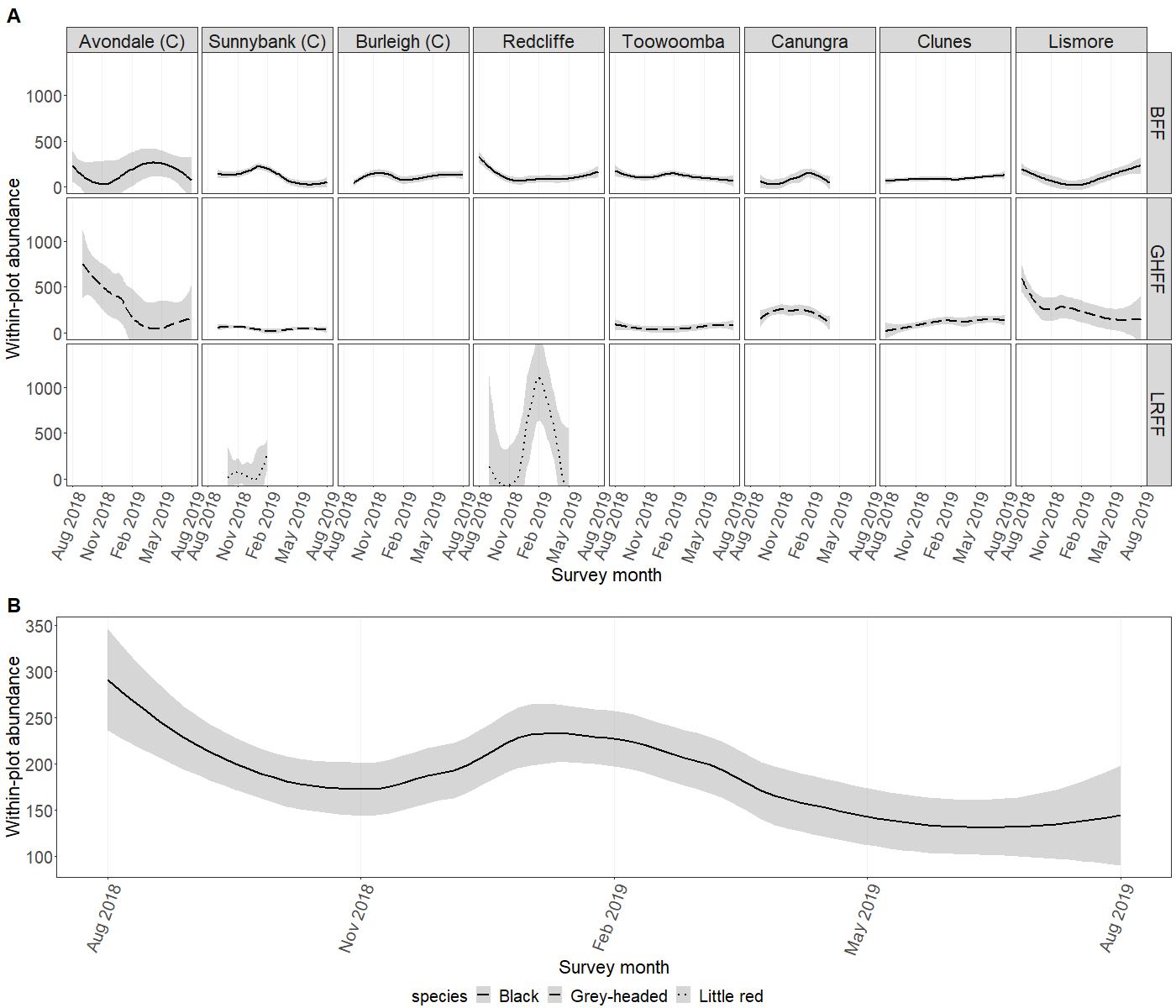


Figure S8: Number of bats per occupied subplot through time, facetted by roost site (columns) and species (rows). Trend line is by loess fit (local polynomial regression fit) with standard error bands (grey shading). A) shows relationship split by roost and B) shows relationship with roosts combined.

Appendix S4: Species density over space and time

Online only interactive images, available at < https://doi.org/10.5061/dryad.g4f4qrfqv >

Appendix S5: Male composition per tree over space and time

Online only interactive images, available at < https://doi.org/10.5061/dryad.g4f4qrfqv >
